# Variants at the 9p21 locus and melanoma risk

**DOI:** 10.1186/1471-2407-13-325

**Published:** 2013-07-02

**Authors:** Livia Maccioni, Panduranga Sivaramakrishna Rachakonda, Justo Lorenzo Bermejo, Dolores Planelles, Celia Requena, Kari Hemminki, Eduardo Nagore, Rajiv Kumar

**Affiliations:** 1 Division of Molecular Genetic Epidemiology, German Cancer Research Centre (DKFZ), Im Neuenheimer Feld 580, D-69120, Heidelberg, Germany; 2 Institute of Medical Biometry and Informatics, University of Heidelberg, Heidelberg, Germany; 3 Laboratory of Histocompatibility-Molecular Biology, Center for Blood Transfusion, Valencia, Spain; 4 Department of Dermatology, Instituto Valenciano de Oncologia, Valencia, Spain; 5 Center for Primary Health Care Research, Lund University, Malmö, Sweden; 6 Universidad Católica de Valencia, Valencia, Spain

## Abstract

**Background:**

The influence of variants at the 9p21 locus on melanoma risk has been reported through investigation of *CDKN2A* variants through candidate gene approach as well as by genome wide association studies (GWAS).

**Methods:**

In the present study we genotyped, 25 SNPs that tag 273 variants on chromosome 9p21 in 837 melanoma cases and 1154 controls from Spain. Ten SNPs were selected based on previous associations, reported in GWAS, with either melanocytic nevi or melanoma risk or both. The other 15 SNPs were selected to fine map the *CDKN2A* gene region.

**Results:**

All the 10 variants selected from the GWAS showed statistically significant association with melanoma risk. Statistically significant association with melanoma risk was also observed for the carriers of the variant T-allele of rs3088440 (540 C>T) at the 3’ UTR of *CDKN2A* gene with an OR 1.52 (95% CI 1.14-2.04). Interaction analysis between risk associated polymorphisms and previously genotyped *MC1R* variants, in the present study, did not show any statistically significant association. Statistical significant association was observed for the interaction between phototypes and the rs10811629 (located in intron 5 of *MTAP)*. The strongest association was observed between the homozygous carrier of the A–allele and phototype II with an OR of 15.93 (95% CI 5.34-47.54).

**Conclusions:**

Our data confirmed the association of different variants at chromosome 9p21 with melanoma risk and we also found an association of a variant with skin phototypes.

## Background

Cutaneous malignant melanoma is one of the cancer types with rapid increase in incidence rates in many Caucasian populations worldwide [1,2]. The risk factors associated with melanoma include environmental sun exposure and genetically determined host factors as skin color, eye color, hair color, freckling and the presence and number of nevi [2-6]. One of the most significant risk factors in melanoma is the family history of the disease. Approximately, 10% of melanoma is associated with familial predisposition with affected first or second-degree relatives. Out of those 20%-40% of the familial melanoma is linked to chromosome 9p21 locus and a proportion of 9p21 linked families carry disease-segregating germline mutations in *cyclin-dependent kinase inhibitor 2A* (*CDKN2A*) gene [7]. Over the years several genome wide association studies (GWAS) have identified a number of low penetrance variants at the locus 9p21 that were associated with risk of either melanoma or cutaneous nevi or both [8-12]. The locus has also been previously investigated through candidate gene approach with particular emphasis on the *CDKN2A* gene for detection of variants associated with risk of melanoma [13-16].

Two GWAS showed the association of a total of 10 single nucleotide polymorphisms (SNPs) at 9p21 locus with either cutaneous nevi or melanoma risk or both. The polymorphisms rs751173, rs1341866 (both intergenic) and rs10811629 located in intron 5 of *methylthioadenosine phosphorylase* (*MTAP*) have been associated with cutaneous nevi while the three intergenic polymorphisms rs4636294, rs2218220, rs1335510 and the two intronic rs10757257 and rs7023329 with cutaneous nevi and melanoma [8,9]. Another study showed association of the imputed intergenic variant rs935053 with melanoma risk. Moreover, stepwise regression analysis of 11 tag SNPs covering a 2 Mb region at the 9p21 locus showed the association between the polymorphism rs1011970 and melanoma [9]. All the polymorphisms except rs1011970 were located in a 132.9 Kb region containing the *methylthioadenosine phosphorylase* (*MTAP*) gene. The polymorphism rs1011970 was located in *antisense non coding RNA* (*ANRIL*) gene*.* The association of the 9p21 locus with melanoma risk was replicated in subsequent GWAS and in a recent meta-analysis [10-12,17].

The association of the polymorphisms 540 C>T (rs3088440) and 500 C>G (rs11515) at the 3’ untranslated region of *CDKN2A* with melanoma risk investigated by candidate gene approach has remained ambiguous. An earlier study based on familial melanoma in an Australian population showed an association of the variant 500 C>G with risk of melanoma [13]. Another study based on sporadic primary melanomas showed statistically significant association of the polymorphism 540 C>T with melanomas but no statistically significant association was reported for the polymorphism 500 C>G. The study showed the linkage disequilibrium of the variant 500 C>G with a polymorphism in intron 1 of the adjacent *cyclin-dependent kinase inhibitor 2B* (*CDKN2B*) gene [14]. However, later studies could not replicate the association of the two polymorphisms with melanoma risk [15,16].

In the present study, we genotyped 25 polymorphisms at 9p21 locus, selected by the use of tagging approach and on the basis of previous reported associated variants in GWAS, in Spanish melanoma cases and matched controls. We confirmed the association between melanoma risk and ten polymorphisms that were previously shown in GWAS to be associated with nevi or melanoma risk or both. Of the other 15 variants selected through tagging SNP approach to fine map *CDKN2A* gene we found an association between the rs3088440 variant and melanoma risk.

## Methods

### Study populations

The present study included 837 Spanish cutaneous melanoma patients. The cutaneous melanoma patients were recruited at the Department of Dermatology, Instituto Valenciano de Oncologia, a referral skin cancer center for the provinces of Valencia, Alicante and Castellón, with ~5 million population. Blood samples from melanoma patients with histopathologically confirmed diagnosis were collected between 2000 and 2007. Clinical and pathological data from the patients were prospectively collected since January 2000 through the review of medical history, personal interviews and clinical examination by expert dermatologists. The patients were followed-up for a median of 88.8 months (95% CI 83.1-94.4). The tumors were classified according to the AJCC stage system for pathological stage and the relative Breslow thickness was reported. The phenotypic characteristics of skin in cases were classified as Fitzpatrick phototypes after examination by a trained dermatologist. Information about hair and eye color was also documented. Spanish control included 1154 ethnically matched disease-free controls-individuals. Disease-free and ethnically matched healthy control subjects were recruited at the Transfusion Center of Valencia, Spain. The phenotypic characteristics of skin in controls were classified as Fitzpatrick phototypes based on a self-reported questionnaire.

All the participants in the study signed an informed consent and the study protocol was approved by institutional ethic board of Instituto Valenciano de Oncologia.

### Selection and genotyping of the polymorphisms

**Figure 1:**
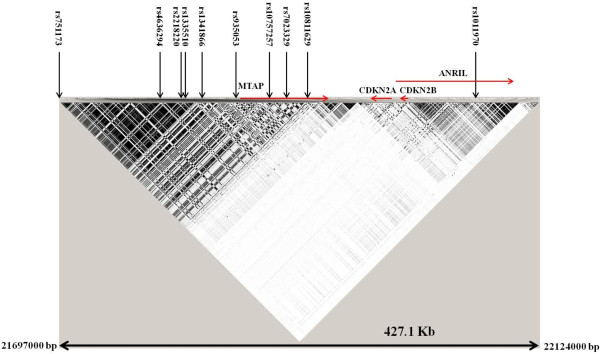
**Linkage disequilibrium map of chromosome 9p21 from 21697000 bp to 22124000 bp.** The linkage disequilibrium map derived from HapMap (release #27) shows the SNPs associated with cutaneous nevi or melanoma risk or both in GWAS.

The polymorphisms on chromosome 9p21 were selected according to two criteria: polymorphisms previously reported to be associated in published GWAS on melanoma and nevi and polymorphisms selected through tagging approach to fine map the *CDKN2A* gene. Ten polymorphisms reported to be associated with melanoma and cutaneous nevi or both were included in the study (Figure 1).

**Figure 2:**
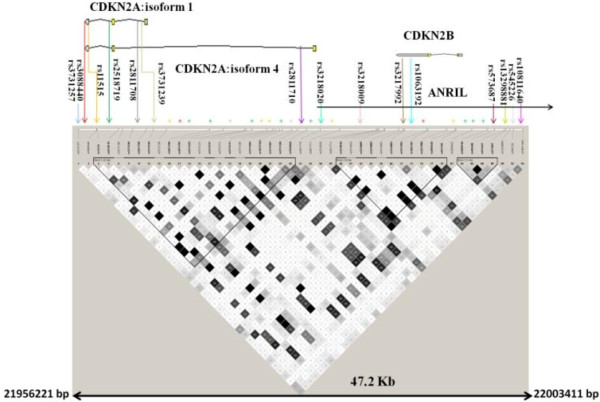
**Linkage disequilibrium map of chromosome 9p21 from 21956221 bp to 22003411 bp.** The map derived from HapMap (release #27) reports the genotyped tagging SNPs.

The selection of the polymorphisms in order to fine map the *CDKN2A* gene was done using data on the Caucasian population from HapMap database (release #27). The 47.2 Kb (chromosome 9: 21956221–22003411; build 36.3) region encompassing *CDKN2A* and the nearby *CDKN2B* was scanned and 12 polymorphisms with a minor allele frequency ≥0.10 were identified using tagger program in Haploview using a minimum r^2^ of 0.8 (Figure 2). A total of 25 polymorphisms were selected on chromosome 9p21 and genotyped in the Spanish melanoma cases and controls (Additional file 1: Table S1).

Genotyping of the selected polymorphisms was carried out using a PCR based allelic discrimination method (KBiosciences UK). The amplification was carried out in a final volume of 4 μl using 5 ng of DNA, 2 μl 2X reaction mix (KBiosciences, UK), 0.06 μl of the solution containing 100 nM of two forward primers specific for two alleles, and one reverse primer mix. The reaction was performed under thermocycling conditions with an initial denaturation at 94°C for 15 minutes, followed by 36–46 cycles of 20s at 94°C and 60s of annealing (temperatures ranging from 60-50°C). Genotypes in amplified products were determined by differences in VIC and FAM fluorescent level in plate read operation on ABI PRISM 7900HT (Applied Biosystems, Foster City, CA) using SDS 1.2 Software. Post operation data were transferred as Microsoft Excel files and converted into genotype informations.

The samples for which genotyping failed in Kaspar assay were randomly sequenced. PCR for sequencing was carried out in a total volume of 10 μl. The reaction contained 10 ng of DNA template, 1.0 μl of 10X reaction buffer, 0.11 mM of each dNTP, 0.20 μM of each primer (Additional file 2: Table S2), 1.50 mM of MgCl_2_ and 0.2 U (TAQ POL) of DNA Taq Polymerase (Genaxxon BioScience GmbH, Germany). Thermocycling conditions used were 94 C for 5 min, followed by 3 cycles of 94°C for 45 seconds, annealing temperature (Additional file 2: Table S2) for 45 seconds, 72°C for 45 seconds followed by additional 35 repeated cycles of 94°C for 30 seconds, annealing temperature (Additional file 2: Table S2) for 30 seconds and 72 C for 45 sec. The final step was 72°C for 5 minutes.

The amplified fragments were checked on 1% agarose gel. PCR product were purified by incubation for 30 minutes at 37 C with 0.75 μl of ExoSAP-IT®(USB Corporation, Cleaveland, OH), followed by an incubation for 15 minutes at 85°C. Cycle sequencing reaction on purified PCR products were performed using specific primers (Additional file 2: Table S2). The sequencing reaction was performed using Big Dye® Terminator Cycle Kit (Applied Biosystem, Foster City, CA). The reaction products were precipitated with isopropanol, washed in 70% ethanol and the precipitate was suspended in 25 μl of water. The 96 well plates containing DNA were loaded on an ABI prism 3100 Genetic analyzer and the data were analyzed using a sequencing program (Applied Biosystem).

### Statistical analysis

Genotype frequencies of all SNPs were tested in control subjects for deviation from Hardy-Weinberg equilibrium using Pearson’s X^2^ test. For association study, 63 patients with melanoma in situ (AJCC stage 0) were excluded from data analysis. The association between cutaneous melanoma and genotype of the polymorphisms was evaluated by odds ratios (OR), 95% confidence intervals (CI) and P values, using logistic regression (SAS version 9.2, SAS institute Inc., NC). Logistic regression models included age and sex as covariates. Analysis was also performed using a model selection that included all covariates.

Gene-gene and gene-host factor interactions were carried out after a model selection based on likelihood ratio tests (LTR). The interaction analysis between the variants that showed association with the risk of melanoma and previously genotyped *melanocortin receptor 1* (*MC1R)* variants was carried out by categorizing both cases and controls into carriers and non-carriers [18]. For *MC1R* categories selected were carriers of any (V60L, D84E, V92M, R151C, I155T, R160W, R163Q and D294H) *MC1R* variants versus non-carriers and analysis was also carried out by grouping red hair color (RHC; D84E, R151C, R160W and D294H) and non-RHC variants separately. All statistical tests were performed in SAS 9.2 software.

Interaction contrast ratio ICR to test departure from additivity (> or < 0) and the multiplicative interaction index MII to test departure from multiplicativity (> or < 1) were also calculated in SAS 9.2. Confidence intervals and the p-values were calculated using bootstrap methods with 10000 simulations (SAS 9.2).

### Bioinformatic analysis

The estimate of linkage disequilibrium between the polymorphisms in Spanish cases and controls combined was performed using SNP_tool package. The converted data were processed in the Haploview software (version 4.2) for linkage disequilibrium estimation.

Analysis of expression quantitative trait loci (eQTL) in the eQTL browser (eqtl.uchicago.edu/Home.html) was performed for the statistically significant associated polymorphisms with melanoma risk and the tagged variants.

## Results

The present study included 837 cutaneous melanoma cases from Spain and 1154 disease-free ethnically matched controls. The cases comprised of 459 women and 378 men with mean and median age of 51.5 (±16.1) and 53 (inter-quartile range: 39–65) years, respectively. Spanish controls were composed of 478 women and 676 men with mean and median age of 45 (±7.3) and 44 (inter-quartile range: 39–50) years, respectively. In cases and controls phenotypic traits like Fitzpatrick phototype, hair and eye color were associated with risk of melanoma in an expected manner as previously reported [19]. The cases and controls were genotyped for a total of 25 polymorphisms that tagged a total of 273 variants at the 9p21 locus. Ten SNPs were selected based on GWAS and an additional 15 were selected to fine map *CDKN2A* gene. Sixty-three melanoma patients with AJCC stage 0 were excluded from data analysis. Information on characteristics of melanoma patients and controls subjects are reported in our previous study [19]. The genotype frequencies for all the variants included in the study were, in controls, in accordance with Hardy-Weinberg equilibrium.

The data analysis showed that of the 25 genotyped variants, all 10 variants selected on the basis of GWAS showed statistically significant association with melanoma risk. Of the 15 variants selected to fine map *CDKN2A* gene, the rs3088440 (3’UTR *CDKN2A*) variant, showed statistically significant association with melanoma risk. In addition the variant T-allele of the tagging rs2811710 variant also showed statistically significant association with the risk of melanoma. None of the remaining 13 tagging polymorphisms showed statistically significant association with the disease risk.

The rs751173 polymorphism located in an intergenic region between the *interferon, epsilon* (*IFNE*) and the *MTAP* genes showed association with a statistical significant increased melanoma risk (Figure 1). The carriers of the variant C-allele were at an increased risk of developing melanoma (OR 1.25, 95% CI 1.01-1.55) (Table 1). The rs751173 polymorphism tagged (with r^2^ ≥0.8) 22 SNPs according to HapMap database. All tagged SNPs were located in the intergenic region between *IFNE* and *MTAP* genes.

**Table 1:** Analysis of genotype data from melanoma cases and controls for polymorphisms on chromosome 9p21 locus

**SNP**	**Cases (%)**	**Controls (%)**	**OR**	**95% CI**	**P value**
**rs751173**					
TT	211 (27.2)	358 (31.1)	Reference		
TC	384 (49.6)	573 (49.7)	1.18	0.94 –1.47	
CC	**179 (23.1)**	**221 (19.2)**	**1.43**	**1.09-1.87**	**0.04**
TC+CC	**563 (72.7)**	**794 (68.9)**	**1.25**	**1.01-1.54**	**0.04**
T allele	806 (52.1)	1286 (56)	Reference		
C allele	**742 (47.9)**	**1015 (44.1)**	**1.19**	**1.04–1.37**	**0.01**
MAF	0.48	0.44			
**rs4636294**					
GG	169 (21.9)	280 (24.3)	Reference		
AG	375 (48.5)	605 (52.5)	1.07	0.84–1.36	
AA	**229 (29.6)**	**268 (23.2)**	**1.41**	**1.08–1.85**	**0.02**
AG+AA	604 (78.1)	873 (75.7)	1.17	0.93–1.47	0.16
G allele	713 (46.1)	1165 (50.5)	Reference		
A allele	**833 (53.9)**	**1141 (49.5)**	**1.19**	**1.04–1.36**	**0.01**
MAF	0.54	0.50			
**rs2218220**					
TT	167 (21.6)	281 (24.4)	Reference		
CT	379 (49)	607 (52.7)	1.09	0.86–1.39	
**CC**	**228 (29.5)**	**264 (22.9)**	**1.45**	**1.11–1.91**	**0.01**
TC+CC	607 (78.4)	871 (75.6)	1.20	0.96–1.51	0.12
T allele	713 (46.1)	1169 (50.7)	Reference		
C allele	**835 (53.9)**	**1135 (49.3)**	**1.21**	**1.05–1.38**	**0.007**
MAF	0.54	0.49			
**rs1335510**					
TT	323 (41.8)	406 (35.4)	Reference		
TG	**352 (45.6)**	**568 (49.5)**	**0.79**	**0.64–0.97**	
GG	**97 (12.5)**	**173 (15.1)**	**0.67**	**0.49–0.91**	**0.01**
TG+GG	**449 (58.2)**	**741 (64.6)**	**0.76**	**0.63–0.93**	**0.007**
T allele	998 (64.6)	1380 (60.2)	Reference		
G allele	**546 (35.4)**	**914 (39.8)**	**0.81**	**0.71–0.94**	**0.004**
MAF	0.35	0.40			
**rs1341866**					
TT	316 (40.8)	398 (34.6)	Reference		
TC	**361 (46.6)**	**576 (50)**	**0.81**	**0.66–0.99**	
CC	**97 (12.5)**	**177 (15.4)**	**0.66**	**0.49–0.90**	**0.02**
TC+CC	**458 (59.2)**	**753 (65.4)**	**0.77**	**0.63–0.94**	**0.01**
T allele	993 (64.2)	1372 (59.6)	Reference		
C allele	**555 (35.9)**	**930 (40.4)**	**0.82**	**0.71–0.94**	**0.004**
MAF	0.36	0.40			
**rs935053**					
AA	172 (22.3)	293 (25.4)	Reference		
AG	373 (48.3)	601 (52)	1.10	0.87–1.40	
GG	**228 (29.5)**	**259 (22.5)**	**1.48**	**1.13–1.94**	**0.009**
AG+GG	601 (78 )	860 (74.6)	1.22	0.97–1.53	0.08
A allele	717 (46.4)	1187 (51.5)	Reference		
G allele	**829 (53.6)**	**1119 (48.5)**	**1.22**	**1.06–1.39**	**0.004**
MAF	0.54	0.49			
**rs10757257**					
GG	320 (41.3)	407 (35.4)	Reference		
GA	**360 (46.5)**	**567 (49.3)**	**0.83**	**0.68–1.02**	
AA	**94 (12.1)**	**177 (15.4)**	**0.65**	**0.48–0.88**	**0.02**
GA+AA	**454 (58.7)**	**744 (64.6)**	**0.79**	**0.65–0.96**	**0.02**
G allele	1000 (64.6)	1381 (60)	Reference		
A allele	**548 (35.4)**	**921 (40)**	**0.81**	**0.71–0.94**	**0.004**
MAF	0.35	0.40			
**rs7023329**					
AA	230 (29.7)	290 (25.2)	Reference		
AG	377 (48.7)	576 (50)	0.84	0.67–1.06	
GG	167 (21.6)	287 (24.9)	0.74	0.56–0.96	0.08
AG+GG	**544 (70.3)**	**863 (74.9)**	**0.81**	**0.65–1.00**	**0.05**
A allele	837 (54.1)	1156 (50.1)	Reference		
G allele	**711 (45.9)**	**1150 (49.9)**	**0.86**	**0.75–0.98**	**0.02**
MAF	0.46	0.50			
**rs10811629**					
AA	313 (40.4)	395 (34.4)	Reference		
AG	**362 (46.8)**	**579 (50.4)**	**0.83**	**0.68–1.03**	
GG	**99 (12.8)**	**174 (15.2)**	**0.70**	**0.52–0.95**	**0.05**
AG+GG	**461 (59.6)**	**753 (65.6)**	**0.80**	**0.66–0.98**	**0.03**
A allele	988 (63.8)	1369 (59.6)	Reference		
G allele	**560 (36.2)**	**927 (40.4)**	**0.84**	**0.73–0.97**	**0.02**
MAF	0.36	0.40			
**rs1011970**					
GG	549 (71.3)	850 (74.1)	Reference		
GT	190 (24.7)	277 (24.2)	1.03	0.82–1.29	
TT	**31 (4)**	**19 (1.7)**	**2.24**	**1.22–4.13**	**0.04**
GT+TT	221 (28.7)	296 (25.8)	1.11	0.89–1.38	0.35
G allele	1288 (83.6)	1977 (86.3)	Reference		
T allele	252 (16.4)	315 (13.7)	1.18	0.97- 1.42	0.10
MAF	0.17	0.14			
***CDKN2A*tagging polymorphysms**					
**SNP**	**Cases (%)**	**Controls (%)**	**OR**	**95% CI**	**P value**
**rs3731257**					
GG	398 (52)	595 (51.8)	Reference		
GA	302 (39.5)	455 (39.6)	0.94	0.77–1.15	
AA	65 (8.5)	98 (8.5)	0.89	0.63–1.27	0.73
GA+AA	367 (48)	553 (48.2)	0.93	0.77–1.13	0.46
G allele	1098 (71.8)	1645 (71.7)	Reference		
A allele	432 (28.2)	651 (28.4)	0.94	0.81–1.09	0.43
MAF	0.28	0.28			
**rs11515**					
GG	538 (69.7)	796 (69.6)	Reference		
GC	214 (27.7)	316 (27.7)	0.96	0.78–1.19	
CC	20 (2.6)	31 (2.7)	0.91	0.50–1.67	0.91
GC +CC	234 (30.3)	347 (30.4)	0.96	0.78–1.18	0.68
G allele	1290 (83.6)	1908 (83.5)	Reference		
C allele	254 (16.5)	378 (16.5)	0.96	0.80–1.15	0.66
MAF	0.17	0.17			
**rs3088440**					
CC	660 (85.4)	1020 (90)	Reference		
CT	**108 (14)**	**108 (9.5)**	**1.52**	**1.13–2.05**	
TT	5 (0.7)	5 (0.4)	1.50	0.40–5.58	**0.02**
CT+TT	**113 (14.6)**	**113 (10)**	**1.52**	**1.14–2.04**	**0.005**
C allele	1428 (92.4)	2148 (94.8)	Reference		
T allele	**118 (7.6)**	**118 (5.2)**	**1.48**	**1.12–1.96**	**0.005**
MAF	0.08	0.05			
**rs2518719**					
AA	541 (70.8)	808 (70.4)	Reference		
AG	206 (27)	320 (27.9)	1.04	0.84–1.30	
GG	17 (2.2)	19 (1.7)	1.58	0.79–3.19	0.42
AG+GG	223 (29.2)	339 (29.6)	1.07	0.87–1.33	0.51
A allele	1288(84.3)	1936 (84.4)	Reference		
G allele	240 (15.7)	358 (15.6)	1.09	0.91–1.31	0.36
MAF	0.16	0.16			
**rs2811708**					
GG	369 (46.8)	563 (49.1)	Reference		
GT	332 (43)	475 (41.5)	1.09	0.89–1.33	
TT	71 (9.2)	108 (9.4)	1.05	0.74–1.48	0.70
GT + TT	403 (52.2)	583 (50.9)	1.08	0.89–1.31	0.42
G allele	1070 (69.3)	1601 (69.9)	Reference		
T allele	474 (30.7)	691 (30.2)	1.05	0.91–1.22	0.52
MAF	0.31	0.30			
**rs3731239**					
AA	356 (46.8)	494 (43)	Reference		
AG	318 (41.8)	536 (46.6)	0.84	0.68–1.03	
GG	86 (11.3)	120 (10.4)	1.08	0.78–1.50	0.13
AG+GG	404 (53.2)	656 (57)	0.88	0.73–1.07	0.20
A allele	1030 (67.8)	1524 (66.3)	Reference		
G allele	490 (32.2)	776 (33.7)	0.97	0.84–1.12	0.65
MAF	0.33	0.34			
**rs2811710**					
CC	286 (37)	450 (39.3)	Reference		
CT	355 (45.9)	542 (47.3)	1.03	0.82–1.27	
TT	132 (17.1)	154 (13.4)	1.40	1.05–1.87	0.06
CT+TT	487 (63)	696 (60.7)	1.10	0.91–1.35	0.30
C allele	927 (60)	1442 (62.9)	Reference		
T allele	**619 (40)**	**850 (37.1)**	**1.15**	**1.00–1.32**	**0.05**
MAF	0.40	0.37			
**rs3218020**					
GG	254 (32.8)	381 (33.2)	Reference		
GA	375 (48.5)	560 (48.8)	0.97	0.78–1.20	
AA	145 (18.7)	206 (17.9)	0.96	0.73–1.27	0.95
GA+AA	520 (67.2)	766 (66.8)	0.97	0.79–1.18	0.74
G allele	883 (57)	1322 (57.6)	Reference		
A allele	665 (43)	972 (42.4)	0.98	0.85–1.12	0.76
MAF	0.43	0.42			
**rs3218009**					
CC	650 (84)	965 (83.8)	Reference		
CG	118 (15.3)	180 (15.6)	1.01	0.77–1.31	
GG	6 (0.8)	6 (0.5)	1.96	0.61–6.29	0.52
CG+GG	124 (16)	186 (16.2)	1.04	0.80–1.34	0.79
C allele	1418 (91.6)	2110 (91.7)	Reference		
G allele	130 (8.4)	192 (8.3)	1.06	0.83–1.35	0.63
MAF	0.08	0.08			
**rs3217992**					
CC	229 (29.9)	341 (29.7)	Reference		
CT	380 (49.7)	565 (49.2)	0.95	0.76–1.19	
TT	156 (20.4)	243 (21.2)	0.89	0.68–1.17	0.72
CT+TT	536 (70.1)	808 (70.3)	0.94	0.76–1.15	0.53
C allele	838 (54.8)	1247 (54.3)	Reference		
T allele	692 (45.2)	1051 (45.7)	0.95	0.83–1.08	0.42
MAF	0.45	0.46			
**rs1063192**					
AA	316 (40.9)	445 (38.8)	Reference		
AG	365 (47.3)	537 (46.9)	0.99	0.80–1.22	
GG	91 (11.8)	164 (14.3)	0.89	0.65–1.20	0.72
AG+GG	456 (59.1)	701 (61.2)	0.97	0.79–1.17	0.72
A allele	997 (64.6)	1427 (62.3)	Reference		
G allele	547 (35.4)	865 (37.7)	0.95	0.83–1.10	0.51
MAF	0.35	0.38			
**rs573687**					
GG	380 (49.3)	532 (46.4)	Reference		
GA	329 (42.7)	501 (43.7)	0.95	0.78–1.17	
AA	62 (8)	114 (9.9)	0.86	0.61–1.22	0.68
GA+AA	391 (50.7)	615 (53.6)	0.94	0.77–1.14	0.50
G allele	1089 (70.6)	1565 (68.2)	Reference		
A allele	453 (29.4)	729 (31.8)	0.94	0.81–1.09	0.40
MAF	0.29	0.32			
**rs13298881**					
TT	565 (73.8)	862 (75.2)	Reference		
TC	183 (23.9)	262 (22.8)	1.14	0.91–1.43	
CC	18 (2.4)	23 (2)	1.26	0.65–2.45	0.46
TC+CC	201 (26.2)	285 (24.9)	1.15	0.92–1.43	0.22
T allele	1313 (85.7)	1986 (86.6)	Reference		
C allele	219 (14.3)	308 (13.4)	1.14	0.93–1.38	0.21
MAF	0.14	0.13			
**rs545226**					
AA	207 (26.8)	300 (26.1)	Reference		
AG	378 (48.9)	577 (50.3)	0.88	0.70–1.11	
GG	188 (24.3)	271 (23.6)	0.93	0.71–1.22	0.55
AG+GG	566 (73.2)	848 (73.9)	0.90	0.72–1.11	0.32
A allele	792 (51.2)	1177 (51.3)	Reference		
G allele	754 (48.8)	1119 (48.7)	0.96	0.84–1.10	0.57
MAF	0.49	0.49			
**rs10811640**					
GG	184 (24.1)	298 (26.1)	Reference		
GT	371 (48.5)	565 (49.4)	1.02	0.81–1.29	
TT	210 (27.5)	280 (24.5)	1.12	0.86–1.46	0.68
GT+TT	581 (76)	845 (73.9)	1.05	0.84–1.32	0.65
G allele	739 (48.3)	1161 (50.8)	Reference		
T allele	791 (51.7)	1125 (49.2)	1.06	0.92–1.21	0.42
MAF	0.52	0.49			

Values in bold indicate statistical significance.

The polymorphisms rs4636294, rs2218220 and rs935053 linked with an r^2^ =1 on HapMap data; showed a similar pattern of association with melanoma risk. All the three variants are located in the intergenic region between *IFNE* and *MTAP* genes (Figure 1). The variant A-allele and the homozygous carriers of the variant allele of the rs4636294 polymorphism showed an increased risk of melanoma with an OR of 1.19 (95% CI 1.04-1.36) and OR of 1.41 (95% CI 1.08-1.85), respectively. Same pattern of association was observed for the rs2218220 and rs935053 polymorphisms (Table 1). According to HapMap data 117 SNPs (including the rs2218220 and rs935053) were tagged by the rs4636294 and all were located in intergenic region between *IFNE* and *MTAP* genes.

Linkage disequilibrium analysis using HapMap data showed that the rs1335510 and rs1341866 polymorphisms also located in the intergenic region between *IFNE* and *MTAP* genes were in complete linkage disequilibrium with an r^2^ =1; an additional polymorphism rs10757257 located in intron 1 of *MTAP* gene was in linkage disequilibrium with r^2^ =0.89 with rs1335510 and rs1341866 (Figure 1). The polymorphism rs1335510 showed a statistically significant decreased risk for the carriers of the G-allele (OR 0.76, 95% CI 0.63-0.93). A similar trend was observed for the carriers of the variant C-allele of the polymorphism rs1341866 and the carriers of the variant A-allele of rs10757257 (Table 1). Among the 37 variants tagged (with r^2^ ≥0.8) by rs1335510 (including rs1341866 and rs10757257), one was located in exon 3 of *MTAP* gene and the other variants were located in intergenic regions or were intronic to *MTAP*. The regions defined by rs751173 and rs4636294-rs2218220-rs935053 were linked to each other with an r^2^ of 0.7. The regions defined by rs751173 and rs1335510-rs1341866 were linked with an r^2^ of 0.41. And finally the regions defined by rs4636294-rs2218220-rs935053 and rs1335510-rs1341866 were in linkage disequilibrium with an r^2^ of 0.65.

The polymorphism rs7023329 located in intron 2 of *MTAP* that tagged 10 additional variants showed a statistical significant association with decreased risk of melanoma (Figure 1). The carriers of the variant G-allele were at a decreased risk of melanoma with an OR 0.81 (95% CI 0.65-1.00; Table 1). A statistically significant decreased risk of melanoma was also observed for the carriers of the G-variant of the polymorphism rs10811629 located in intron 5 of *MTAP* gene (OR 0.80, 95% CI 0.66-0.98) (Table 1).

No statistically significant association with melanoma was observed for the carrier of the variant T-allele of the rs1011970 polymorphism located in intron 9 of *ANRIL* gene (Figure 1). However, a statistically significant association with increased melanoma risk was observed for the homozygous carriers of the variant T-allele of the rs1011970 polymorphism (OR 2.24, 95% CI 1.22-4.13; Table 1).

The polymorphism rs3088440 located at the 3’UTR of *CDKN2A* gene, tagged six variants located in intronic regions of *CDKN2A/ARF*, *ANRIL* and 3’UTR of *CDKNB* gene as well as intergenic variants between *MTAP* and *CDKN2A* genes. The carriers of the variant T-allele of the rs3088440 variant showed a statistically significant increased risk of melanoma with an OR of 1.52 (95% CI 1.14-2.04) (Figure 2; Table 1). In addition statistically significant association with melanoma risk was also observed for the variant T-allele of the polymorphism rs2811710 located in intron 1 of *CDKN2A/ARF* (OR 1.15, 95% CI 1.00-1.32) (Figure 2; Table 1). The use of logistic regression model that, besides all covariates like age and gender, included all the 25 genotyped polymorphisms on chromosome 9p21 showed retention of statistical significant association for the rs935053 and rs3088440 polymorphisms.

### Associated variants and expression quantitative trait loci analysis on chromosome 9p21

All the associated polymorphisms and tagged SNPs were analyzed for being eQTL. Polymorphisms that tagged to rs751173, rs4636294 (and the linked rs2218220 and rs935053), rs1335510 (and the linked rs1341866 and rs10757257), rs7023329 and rs2811710 were reported as eQTL. All the eQTL variants were *cis* acting. The genes with affected expressions were *MTAP* and *CDKN2B* (Additional file 3: Table S3).

### Linkage disequilibrium analysis

**Figure 3:**
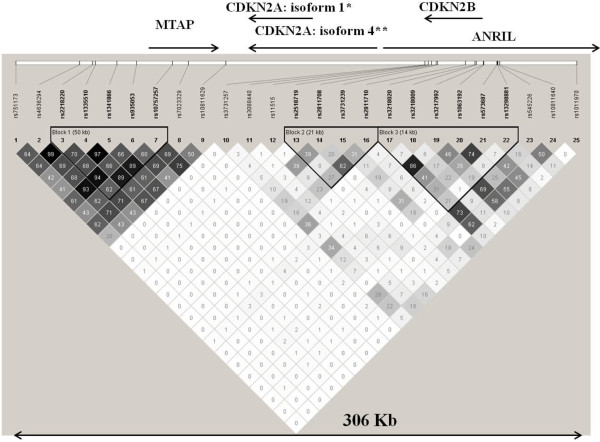
**Linkage disequilibrium map of the genotyped polymorphisms on chromosome 9p21 in the Spanish population.** *CDKN2A isoform 1 encodes p16^INK4A^ ;**CDKN2A isoform 4 (*CDKN2A/ARF*) encodes p14^ARF^.

Linkage disequilibrium analysis was performed in the Spanish population (cases and controls were analyzed together). We observed that the linkage disequilibrium with r^2^ ≥0.8 reported in HapMap was replicated in our data for rs4636294, rs2218220 and rs935053 as well as for rs1335510, rs1341866 and rs10757257 variants. An additional linkage disequilibrium not reported in HapMap data was observed between the rs3218020 and rs3217992. The linkage disequilibrium between the two variants was r^2^ of 0.86 (Figure 3).

### Interaction of associated variants with *MC1R* variants and host risk factors

Gene-gene interaction analysis was performed only for the polymorphisms that showed statistically significant association with melanoma risk. Interaction analysis with *MC1R* variants did not showed any statistically significant association (Additional file 4: Table S4). A statistical significant interaction for melanoma risk was observed between phototypes and the rs10811629 (Additional file 5: Table S5). Strongest interaction was observed between the homozygotes for the A–allele and phototype II with OR of 15.93 (95% CI 5.34-47.54) when compared to the carrier of the variant G-allele with phototype V. A statistically significant interaction was also observed between carrier of the A-allele and phototype I (OR 8.43; 95% CI 2.51-28.33); phototype III (OR 5.83; 95% CI 1.99-17.11) and phototype IV (OR 7.79; 95% CI 2.59-23.43; Additional file 5: Table S5). No other statistically significant interaction was observed between the genotyped polymorphisms and other host risk factors.

The strongest gene-host factor interaction observed between homozygotes for the A–allele and phototype II was also tested for departure from additivity and multiplicativity. No departure from additivity (ICR 9.07; 95% CI −1.54-32.81; P value 0.09) and multiplicativity was observed (MII 1.97; 95% CI 0. 67–5.85; P value 0.15).

## Discussion

**Figure 4:**
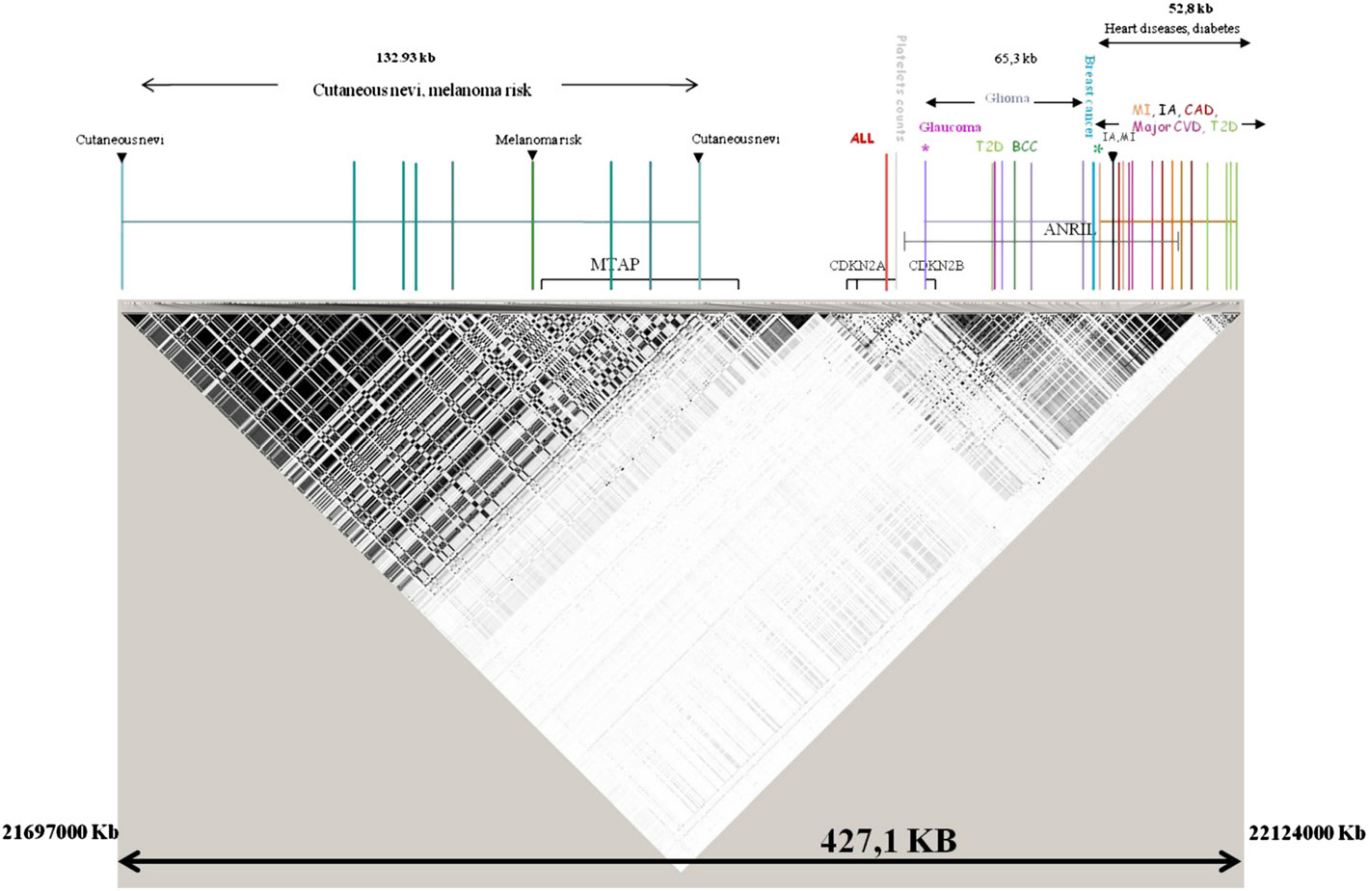
**Linkage disequilibrium map of chromosome 9p21 from 21697000 bp to 22124000 bp.** The map derived from HapMap (release #27) shows the SNPs associated in GWAS with several cancers including melanoma and other diseases. T2D Type II diabetes, MI myocardialinfarction, CVD cardiovascular disease, CAD coronary artery disease, BCC Basal cell carcinoma, IA Intracranial aneurism, ALL Acute lymphoblastic leukemia*.*

Several GWAS over the years have shown association between the 9p21 locus and several cancers including glioma, basal cell carcinoma of skin and several diseases like type-2 diabetes (Figure 4) [20-31]. In particular two GWAS reported the association of nine variants with one additional variant in the replication phase with risk of either cutaneous nevi or melanoma or both [8,9]. In the present study we could replicate the association between the variants reported in GWAS with melanoma risk. In addition we also genotyped 15 variants tagging the *CDKN2A* gene and we observed a statistically significant association with melanoma risk for the previously inconsistently associated rs3088440 variant.

All the variants selected from the two GWAS associated with melanoma risk excluding rs1011970 were located in a 132.9 Kb region including *MTAP* gene. In this region the associated variants and the 187 tagged variants were located in intergenic region telomeric to *MTAP* gene or intronic to *MTAP* gene; only one variant was located in exon 3 of *MTAP*. Interestingly a study reported that the *MTAP* variant rs10757257 was associated with increased risk of superficial spreading/nodular melanoma but not with lentigo malignant melanoma that is the subtype associated with chronic sun exposure. Those results suggested that the relationship between MTAP and melanoma could be subtype-specific [32]. *MTAP* gene plays an important role in polyamine metabolism and the salvage of adenine and methionine. MTAP is reported to show low expression in malignant melanoma cells and conversely its substrate i.e., 5′-deoxy-5’-(methylthio) adenosine (MTA) has higher expression in melanoma cells compared to normal melanocytes. The increased level of MTA has been related to an increased invasive potential and vascuologenesis [33,34]. Loss of *MTAP* expression in melanoma is mainly due to hypermethylation of the promoter region. It was suggested that loss of MTAP expression in malignant melanomas might have an impact on therapeutic success by compromising tumor response to interferon treatment through its impact on STAT1 activity [33,35,36].

On the basis of the allelic P values we observed that all the GWAS variants except rs1011970 (intron 9 *ANRIL*) showed statistically significant association. The polymorphism rs1011970 however showed statistically significant association with melanoma risk only for the homozygotes of the variant T-allele. The polymorphism rs1011970 reported to be associated with melanoma was also associated with breast cancer risk modulation in a GWAS [9,37]. *ANRIL* gene located in the antisense orientation to that of the *CDKN2A* and *CDKN2B* genes consists of 19 exons. *ANRIL* gene is transcribed into several different splice variants of non-coding-RNA [38-40]. The first exon of *ANRIL* gene overlaps the two exons of *CDKN2B* gene. A study observed that silencing of *ANRIL* resulted in 8-fold increase of *CDKN2B* expression suggesting a negative regulation. The study suggested that ANRIL binds to and recruits polycomb repression complex 2 (PCR2) to repression of *CDKN2B*[41]. In addition, the 5’ end of the first exon of the ANRIL gene is located ~300 bp upstream of the transcription start site of *CDKN2A/ARF* gene [39]. The two genes are physically linked by the intergenic region and co-regulated by E2F1 [40]. Interestingly several GWAS identified the association of genetic variants in the *ANRIL* gene with several cancer and diseases including basal cell carcinoma, breast cancer, glioma, coronary disease, intracranial aneurysm and also type 2 diabetes [39]. The association of variants within *ANRIL* gene and several cancers suggests a plausible involvement of the gene in melanoma etiology. The role of *CDKN2A* on melanogenesis is mainly explained by the presence of germline mutations in a proportion of familial melanoma [7]. However, the influence of the polymorphisms rs11515 (500 G>C), rs3088440 (540 C>T) at the 3′ UTR of the gene and the coding variant A148T located in exon 2 on melanoma risk has been extensively investigated in different studies over the years by candidate gene approach and meta-analysis. The association of the *CDKN2A* polymorphisms with melanoma risk reported in those studies lack consistency [14-17,42,43]. In the present study we observed for the carriers of the T –allele of the polymorphism rs3088440 a statistically significant association with melanoma risk. The associated variant tagged a total of 6 SNPs of which 3 were located in intergenic region and the other three in intron 1 of *CDKN2A*, 3’UTR of *CDKN2B* and intron 3 of *ANRIL.* Association with melanoma risk was also observed for the variant T-allele of rs2811710 located in intron 1 of *CDKN2A/ARF*. The associated variant tagged (with r^2^ ≥0.80) three polymorphisms, all located in a intergenic region between the *MTAP* and *CDKN2A* genes. The effect of the rs3088440 at the 3′UTR and of the intronic variants on *CDKN2A* cannot be ruled out.

The majority of the variants associated with melanoma risk and also most of the tagged variants in the present study were located in intergenic or intronic regions. The role of the associated and tagged variants in the melanoma etiology still needs to be clarified. However the intronic variants can possibly affect the expression of respective genes as being part of enhancer elements through binding of transcription factors as reported in previous studies [44,45]. Intronic variants can also affect gene splicing. Although some intronic polymorphisms are not located at the splice junctions, they can still act so as to change the splicing phenotype as a consequence of their being located within an intron splice enhancer or branchpoint site, or by activating a cryptic splice site as reported in several studies [46]. Alteration in gene splicing and consequent alteration in protein structure could lead to disease development [47]. Intronic and intergenic polymorphisms can be expression quantitative trait loci (eQTLs) and can alter gene expression possibly explaining the observed association with melanoma risk. Moreover, the possibility of the associated variants being in linkage disequilibrium with rare functional variants cannot be precluded.

We investigated the associated and linked variants to be eQTL using the eQTL browser eqtl.uchicago.edu/Home.html. We observed that variants linked to the associated intergenic rs751173, rs4636294, rs2218220, rs935053, rs1335510 and the intronic rs7023329 and rs2811710 polymorphisms were eQTL and showed regulation of *MTAP* and *CDKN2B* genes. *CDKN2B* gene encodes for the tumor suppressor protein p15^INK4B^ that it is homologous to p16^INK4A^[48]. Several lines of evidence indicate that p15^INK4B^ inactivation is not significant in the development of melanoma. First, despite its frequent co-deletion with *CDKN2A*, germline or acquired mutations that target *CDKN2B* exclusively have not been observed [49,50]. Second, *CDKN2B*-null mice showed a minimal cancer-prone phenotype and do not develop melanoma [49,51].

In the present study we observed effect of the associated variants on chromosome 9p21 independent of *MC1R* variants on melanoma risk. However due to the limited size of the present study we could not exclude a possible epistatic interaction of *MC1R* variants and 9p21 locus variants on melanoma risk. We also investigated presence of epistatic interaction between associated variants and host risk factor and we observed an interaction between phototype and the rs10811629 (intron 5 *MTAP*) variant. The highest increased risk was observed for the homozygotes of the risk A-allele with phototype II compared to the reference carrier of the G-allele and phototype V. No other epistatic interaction was observed in the gene-host risk factors analysis. It may be pointed out that self-reporting of phenotype in controls could be of questionable precision, therefore the interpretation of the detected association between the variant and the phototype desires a cautious interpretation.

## Conclusions

In conclusion, the present study on variants at 9p21 locus replicated the association with melanoma risk of the GWAS selected variants. In addition for the variants selected for tagging *CDKN2A* gene; the strongest association with melanoma risk was observed for rs3088440 (3′UTR of *CDKN2A* gene) that has been inconsistently associated with melanoma in previous studies [14-16]. In the present study we also observed an epistatic interaction between the polymorphism rs10811629 (intron 5 *MTAP*) and phototype. The effect of the associated or tagged variants on the respective genes and their role as eQTL on other genes could not be ruled out. However it is biologically plausible that variants affecting the genes *MTAP*, *CDKN2A* and *ANRIL* could have an important role in melanoma etiology. Further investigations are needed to clarify the role of *CDKN2B* in melanoma disease and how the variants affecting the gene could be related to melanoma risk. Our finding together with previous GWAS provided evidence of an involvement of variants at the 9p21 locus and melanoma risk. This represents a unique study that investigated a large homogenous case–control population for the association of tagging polymorphisms on chromosome 9p21 discovered through genome wide association studies and polymorphisms at the CDKN2A locus with melanoma risk and association with host factors.

## Abbreviations

CDKN2A: Cyclin-dependent kinase inhibitor 2A; GWAS: Genome wide association studies; CDK4: Cyclin-dependent kinase 4; Rb: Retinoblastoma; MDM2: Mouse double minute −2; MTAP: Methylthioadenosine phosphorylase; SNP: Single nucleotide polymorphism; ANRIL: Antisense non coding RNA; CDKN2B: Cyclin-dependent kinase inhibitor 2B; AJCC: American Joint Committee on Cancer; OR: Odds ratios; 95% CI: 95% confidence intervals; LTR: Likelihood ratio tests; MC1R: Melanocortin receptor 1; eQTL: expression quantitative trait loci; IFNE: Interferon, epsilon; PCR2: Polycomb repression complex 2; E2F1: E2F transcription factor 1; MTA: 5’-deoxy-5’-(methylthio) adenosine; STAT1: Signal transducer and activator of transcription 1.

## Competing interests

The authors declare that they have no competing interest.

## Authors’ contributions

LM genotyped and performed statistical analysis of the polymorphisms and wrote the paper. PSR extracted the DNA from blood for the healthy donors and genotyped. JLB supervised statistical analysis for gene-phenotypic traits interaction. DP and CR provided samples and data. KH supervised the study. EN provided the samples for the study and clinical data. RK supervised and coordinated the study; revised the manuscript. All authors contributed to the manuscript finalization. All authors read and approved the final manuscript.

## Pre-publication history

The pre-publication history for this paper can be accessed here:

http://www.biomedcentral.com/1471-2407/13/325/prepub

## Supplementary Material

Additional file 1: Table S1 Detailed information of genotyped single nucleotide polymorphisms on chromosome 9p21. Click here for file

Additional file 2: Table S2 Primer sequences and conditions for PCR. Click here for file

Additional file 3: Table S3 eQTL for the polymorphisms tagged (r^2^ ≥0.8) by rs751173, rs4636294, rs2218220, rs935053, rs1335510, rs7023329, rs2811710. Click here for file

Additional file 4: Table S4 Effect of interaction between the polymorphisms rs751173, rs4636294, rs2218220, rs1335510, rs1341866, rs935053, rs10757257, rs7023329, rs10811629, rs1011970, rs3088440, rs2811710 and *MC1R* variants on melanoma risk. Click here for file

Additional file 5: Table S5 Effect of interaction between the polymorphisms rs751173, rs4636294, rs2218220, rs1335510, rs1341866, rs935053, rs10757257, rs7023329, rs10811629, rs1011970, rs3088440, rs2811710 and phenotypic traits on melanoma risk. Click here for file
