# Supplementary material for: Variants at the 9p21 locus and melanoma risk
Source: BMC Cancer. 2013 Jul 2;13:325. doi: 10.1186/1471-2407-13-325 (PMC3702420; doi:10.1186/1471-2407-13-325)
Supplement: Additional file 1: Table S1 — Detailed information of genotyped single nucleotide polymorphisms on chromosome 9p21. [file 1471-2407-13-325-S1.docx]

Additional file 1. Detailed information of genotyped single nucleotide polymorphisms on chromosome 9p21

| **SNP selected from GWAS** | **chromosome position** | **nucleotidic change** | **gene location** | **total tagged in HapMap** |
| --- | --- | --- | --- | --- |
| rs751173 | 21697372 | T>C |  | 22 |
| rs4636294 | 21727803 | A>G |  | 117 |
| rs2218220 | 21746089 | C>T |  | 117 |
| rs1335510 | 21747803 | T>G |  | 37 |
| rs1341866 | 21761241 | T>C |  | 37 |
| rs935053 | 21773922 | A>G |  | 117 |
| rs10757257 | 21796564 | G>A | intron 1 *MTAP* | 37 |
| rs7023329 | 21806528 | A>G | intron 2 *MTAP* | 10 |
| rs10811629 | 21830298 | A>G | intron 5 *MTAP* | 1 |
| rs1011970 | 22052134 | G>T | intron 9 *ANRIL* | 0 |
| **SNP from tagging approach** |  |  |  |  |
| rs3731257 | 21956221 | G>A |  | 0 |
| rs3088440 | 21958159 | C>T | 3' UTR *CDKN2A* | 6 |
| rs11515 | 21958199 | G>C | 3' UTR *CDKN2A* | 9 |
| rs2518719 | 21960427 | A>G | intron 2 *CDKN2A* | 9 |
| rs2811708 | 21963422 | G>T | intron 1 *CDKN2A* | 1 |
| rs3731239 | 21964218 | A>G | intron 1 *CDKN2A* | 0 |
| rs2811710 | 21981923 | C>T | intron 1 *CDKN2A/ARF* | 3 |
| rs3218020 | 21987872 | G>A | intron 1 *ANRIL* | 0 |
| rs3218009 | 21988757 | C>G | intron 1 *ANRIL* | 0 |
| rs3217992 | 21993223 | C>T | 3' UTR *CDKN2B* | 2 |
| rs1063192 | 21993367 | A>G | 3' UTR *CDKN2B* | 18 |
| rs573687 | 22001642 | G>A | intron 1 *ANRIL* | 18 |
| rs13298881 | 22002051 | T>C | intron 1 *ANRIL* | 1 |
| rs545226 | 22002422 | A>G | intron 1 *ANRIL* | 0 |
| rs10811640 | 22003411 | G>T | intron 1 *ANRIL* | 19 |
