# Supplementary material for: Variants at the 9p21 locus and melanoma risk
Source: BMC Cancer. 2013 Jul 2;13:325. doi: 10.1186/1471-2407-13-325 (PMC3702420; doi:10.1186/1471-2407-13-325)
Supplement: Additional file 2: Table S2 — Primer sequences and conditions for PCR. [file 1471-2407-13-325-S2.docx]

Additional file 2: Primer sequences and conditions for PCR.

| **SNP** | **Primers**  **5‘-3‘** | **PCR product size** | **Annealing temperature** |
| --- | --- | --- | --- |
| **SNPs chromosome 9p21** |  |  |  |
| rs751173 | CGGCCTCCAAAGACAACATAG  GCCTCTGGGGAAAAGATGCT | 270 bp | 56 |
| rs4636294 | GCCTTCAAAGAACCAACA  TGTCTGTCTAACTGCCATTTC | 313 bp | 53 |
| rs2218220 | ACATTTCCTCCTTCCCCACTT  CCATTGTTGCCTCACCCATAG | 302 bp | 56 |
| rs1335510 | CAAACATCTGCCCTAGAGAA  TTAGAAGACTGGGACTCCAAT | 372 bp | 54 |
| rs1341866 | ACAATGAAGCCTAAGCCCTAT  CCTGCCAGTGTGAGAGC | 251 bp | 55 |
| rs935053 | GCCATTATATGTAGCAAGAAT  GAGATTGATAAAGATGGAGGT | 375 bp | 52 |
| rs10757257 | GCCAAGGAGGGTGAGTGTAG  TGTATCTGGGTGCCAAAGACT | 373 bp | 60 |
| rs7023329 | CTGGGGTACTCGTTACAAATA  CCAAAATTAAGGCATCAGA | 286 bp | 53 |
| rs10811629 | AAGAAAAGACAGGGCTAACG  TTTCACATAACCCCCTAAATC | 314 bp | 55 |
| rs1011970 | AAAATATGTAGGCTTGTGC ACCCTTTTGAAAACTACTAAC | 427 bp | 51 |
| rs2518719 | AATGTTGGCAGTTTCAGCAGA  GGCCAGTTGCATCCACTTAC | 440 bp | 59 |
| rs2811708 | TTAGGATTCTAAGCCAACAT  CATGGGTAACTTAGCATCTCT | 203 bp | 50 |
| rs2811710 | TGAACTAACGTGGAATAATAG  CCACGTTACCAGTTTCTTA | 411 bp | 50 |
| rs3218020 | AGCCTAGCCAAGTTGACAT  ATGGCATTCCTCTGAAATAAG | 210 bp | 53 |
| rs3218009 | AATAGGGAGTGAGTTATCTGT  GGTTTTGAGTTTGAGCAT | 324 bp | 51 |
| rs3217992 | ACAATGGAGCTAGAAGCAG  AGTTGCATTATACTGGGTCAT | 434 bp | 50 |
| rs1063192 | AATGGAGCTAGAAGCAG  AAATTATCCCTTGAAATAGAT | 408 bp | 50 |
| rs573687 | TGGGTATAACTTTTGCGACT  GCCACTCATTCCCTTCTACT | 480 bp | 53 |
| rs13298881 | TAATGGTAAATTGGCATAGAA  CGACCTCAAGGGTGAT | 259 bp | 53 |
| rs10811640 | ATTGTGAGGTATTGGGCTTAG  GGCCGAGGTGAAAGAA | 470 bp | 54 |
