# Supplementary material for: Variants at the 9p21 locus and melanoma risk
Source: BMC Cancer. 2013 Jul 2;13:325. doi: 10.1186/1471-2407-13-325 (PMC3702420; doi:10.1186/1471-2407-13-325)
Supplement: Additional file 3: Table S3 — eQTL for the polymorphisms tagged (r2 ≥0.8) by rs751173, rs4636294, rs2218220, rs935053, rs1335510, rs7023329, rs2811710. [file 1471-2407-13-325-S3.docx]

Additional file 3. eQTL for the polymorphisms tagged (r^2^ ≥0.8) by rs751173, rs4636294, rs2218220, rs935053, rs1335510, rs7023329, rs2811710

| **SNP** | **Tagging variants as eQTL** | **Gene** |
| --- | --- | --- |
| rs751173 |  |  |
|  | rs7874607 | *CDKN2B* |
|  | rs10811584 | *MTAP* |
|  | rs10811586 | *MTAP* |
|  | rs10811590 | *MTAP* |
|  | rs1452658 | *MTAP* |
|  | rs1889680 | *MTAP* |
|  | rs41517746 | *MTAP* |
|  | rs6475552 | *MTAP* |
|  | rs7042962 | *CDKN2B* |
|  | rs7848524 | *MTAP* |
|  | rs7874607 | *CDKN2B* |
| rs4636294, rs2218220, rs935053 |  |  |
|  | rs4636294 | *MTAP* |
|  | rs7847574 | *MTAP* |
|  | rs10757238 | *MTAP* |
|  | rs1335508 | *MTAP* |
|  | rs1345022 | *MTAP* |
|  | rs1414237 | *MTAP* |
|  | rs1414242 | *MTAP* |
|  | rs2184551 | *MTAP* |
|  | rs2891159 | *MTAP* |
|  | rs4341236 | *MTAP* |
|  | rs6475566 | *MTAP* |
|  | rs7021538 | *MTAP* |
|  | rs7846749 | *MTAP* |
|  | rs7847574 | *MTAP* |
|  | rs7866540 | *MTAP* |
|  | rs9886831 | *MTAP* |
| rs1335510 |  |  |
|  | rs10114559 | *MTAP* |
|  | rs10123637 | *MTAP* |
|  | rs10757257 | *MTAP* |
|  | rs10965117 | *MTAP* |
|  | rs1335510 | *MTAP* |
|  | rs1335511 | *MTAP* |
|  | rs1345023 | *MTAP* |
|  | rs1345024 | *MTAP* |
|  | rs2165409 | *MTAP* |
|  | rs3900787 | *MTAP* |
|  | rs4364717 | *MTAP* |
|  | rs7023954 | *MTAP* |
|  | rs7038523 | *MTAP* |
|  | rs7049092 | *MTAP* |
| rs7023329 |  |  |
|  | rs3928894 | *MTAP* |
| rs2811710 |  |  |
|  | rs2811710 | *CDKN2B* |
|  | rs10757261 | *CDKN2B* |
