# Supplementary material for: Variants at the 9p21 locus and melanoma risk
Source: BMC Cancer. 2013 Jul 2;13:325. doi: 10.1186/1471-2407-13-325 (PMC3702420; doi:10.1186/1471-2407-13-325)
Supplement: Additional file 4: Table S4 — Effect of interaction between the polymorphisms rs751173, rs4636294, rs2218220, rs1335510, rs1341866, rs935053, rs10757257, rs7023329, rs10811629, rs1011970, rs3088440, rs2811710 and MC1R variants on melanoma risk. [file 1471-2407-13-325-S4.docx]

Additional file 4. Effect of interaction between the polymorphisms rs751173, rs4636294, rs2218220, rs1335510, rs1341866, rs935053, rs10757257, rs7023329, rs10811629, rs1011970, rs3088440 , rs2811710 and *MC1R* variants on melanoma risk

| **Variable** | **SNP** | **Cases (%)** | **Controls (%)** | **OR** | **CI 95%** | **P value** |
| --- | --- | --- | --- | --- | --- | --- |
|  | **rs751173** |  |  |  |  |  |
| MC1R |  |  |  |  |  |  |
| No variants | TT+TC | 207 (28.8) | 412 (40.3) | Reference |  |  |
|  | CC | 63 (8.8 ) | 96 (9.4) | 1.40 | 0.96–2.04 |  |
| Any variants | TT+TC | 343 (47.8) | 409 (40) | 1.81 | 1.43–2.28 |  |
|  | CC | 105 (14.6) | 106 (10.4) | 2.13 | 1.53–2.98 | < 0.0001 |
| MC1R*rs751173 |  |  |  |  |  | 0.50 |
| MC1R |  |  |  |  |  |  |
| No variants | TT+TC | 207 (28.8) | 412 (40.3) | Reference |  |  |
|  | CC | 63 (8.8 ) | 96 (9.4) | 1.40 | 0.96–2.03 |  |
| No RHC variants | TT+TC | 217 (30.2) | 300 (29.3) | 1.59 | 1.23–2.05 |  |
|  | CC | 64 (8.9) | 77 (7.5) | 1.75 | 1.19–2.59 |  |
| RHC variants | TT+TC | 126 (17.6) | 109 (10.7) | 2.39 | 1.74–3.30 |  |
|  | CC | 41 (5.7) | 29 (2.8) | 3.21 | 1.88–5.48 | < 0.0001 |
| MC1R*rs751173 |  |  |  |  |  | 0.68 |
|  | **rs4636294** |  |  |  |  |  |
| MC1R |  |  |  |  |  |  |
| No variants | GG+GA | 196 (27.3) | 389 (38) | Reference |  |  |
|  | AA | 73 (10.2) | 120 (11.7) | 1.22 | 0.85–1.73 |  |
| Any variants | GG+GA | 310 (43.2) | 391 (38.2) | 1.69 | 1.33–2.15 |  |
|  | AA | 138 (19.3) | 124 (12.1) | 2.30 | 1.68–3.14 | < 0.0001 |
| MC1R*rs4636294 |  |  |  |  |  | 0.64 |
| MC1R |  |  |  |  |  |  |
| No variants | GG+GA | 196 (27.3) | 389 (38) | Reference |  |  |
|  | AA | 73 (10.2) | 120 (11.7) | 1.22 | 0.85–1.73 |  |
| No RHC variants | GG+GA | 201 (28) | 290 (28.3) | 1.49 | 1.15–1.94 |  |
|  | AA | 80 (11.2) | 87 (8.5) | 1.91 | 1.33–2.74 |  |
| RHC variants | GG+GA | 109 (15.2) | 101 (9.9) | 2.25 | 1.61–3.15 |  |
|  | AA | 58 (8.1) | 37 (3.6) | 3.23 | 2.02–5.16 | < 0.0001 |
| MC1R* rs4636294 |  |  |  |  |  | 0.87 |
|  | **rs2218220** |  |  |  |  |  |
| MC1R |  |  |  |  |  |  |
| No variants | TT+CT | 197 (27.4) | 393 (38.4) | Reference |  |  |
|  | CC | 73 (10.2) | 117 (11.4) | 1.26 | 0.88–1.79 |  |
| Any variants | TT+CT | 311 (43.3) | 391 (38.2) | 1.71 | 1.35–2.17 |  |
|  | CC | 137 (19.1) | 122 (11.9) | 2.33 | 1.71–3.19 | < 0.0001 |
| MC1R*rs2218220 |  |  |  |  |  | 0.73 |
| MC1R |  |  |  |  |  |  |
| No variants | TT+CT | 197 (27.4) | 393 (38.4) | Reference |  |  |
|  | CC | 73 (10.2) | 117 (11.4) | 1.26 | 0.88–1.79 |  |
| No RHC variants | TT+CT | 202 (28.1) | 291 (28.5) | 1.51 | 1.16–1.96 |  |
|  | CC | 79 (11) | 86 (8.4) | 1.92 | 1.33–2.76 |  |
| RHC variants | TT+CT | 109 (15.2) | 100 (9.8) | 2.28 | 1.63–3.19 |  |
|  | CC | 58 (8.1) | 36 (3.5) | 3.34 | 2.08–5.36 | < 0.0001 |
| MC1R*rs2218220 |  |  |  |  |  | 0.88 |
|  | **rs1335510** |  |  |  |  |  |
| MC1R |  |  |  |  |  |  |
| No variants | GG+TG | 159 (22.1) | 328 (32.2) | Reference |  |  |
|  | TT | 111 (15.5) | 177 (17.4) | 1.29 | 0.94–1.77 |  |
| Any variants | GG+TG | 262 (36.5) | 325 (31.9) | 1.77 | 1.36–2.30 |  |
|  | TT | 186 (25.9) | 189 (18.6) | 2.16 | 1.61–2.89 | < 0.0001 |
| MC1R*rs1335510 |  |  |  |  |  | 0.81 |
| MC1R |  |  |  |  |  |  |
| No variants | GG+TG | 159 (22.1) | 328 (32.2) | Reference |  |  |
|  | TT | 111 (15.5) | 177 (17.4) | 1.29 | 0.94–1.77 |  |
| No RHC variants | GG+TG | 170 (23.7) | 246 (24.1) | 1.52 | 1.15–2.03 |  |
|  | TT | 111 (15.5) | 131 (12.9) | 1.89 | 1.36–2.63 |  |
| RHC variants | GG+TG | 92 (12.8) | 79 (7.8) | 2.52 | 1.74–3.67 |  |
|  | TT | 75 (10.5) | 58 (5.7) | 2.76 | 1.83–4.15 | < 0.0001 |
| MC1R*rs1335510 |  |  |  |  |  | 0.85 |
|  | **rs1341866** |  |  |  |  |  |
| MC1R |  |  |  |  |  |  |
| No variants | CC+TC | 160 (22.3) | 335 (32.8) | Reference |  |  |
|  | TT | 110 (15.3) | 173 (16.9) | 1.32 | 0.96–1.81 |  |
| Any variants | CC+TC | 268 (37.3) | 329 (32.2) | 1.82 | 1.40–2.36 |  |
|  | TT | 180 (25.1) | 185 (18.1) | 2.16 | 1.61–2.89 | < 0.0001 |
| MC1R*rs1341866 |  |  |  |  |  | 0.62 |
| MC1R |  |  |  |  |  |  |
| No variants | CC+TC | 160 (22.3) | 335 (32.8) | Reference |  |  |
|  | TT | 110 (15.3) | 173 (16.9) | 1.32 | 0.96–1.81 |  |
| No RHC variants | CC+TC | 175 (24.4) | 249 (24.4) | 1.57 | 1.19–2.09 |  |
|  | TT | 106 (14.8) | 127 (12.4) | 1.88 | 1.35–2.63 |  |
| RHC variants | CC+TC | 93 (13) | 80 (7.8) | 2.56 | 1.77–3.71 |  |
|  | TT | 74 (10.3) | 58 (5.7) | 2.75 | 1.82–4.15 | < 0.0001 |
| MC1R*rs1341866 |  |  |  |  |  | 0.77 |
|  | **rs935053** |  |  |  |  |  |
| MC1R |  |  |  |  |  |  |
| No variants | AA+AG | 196 (27.3) | 395 (38.6) | Reference |  |  |
|  | GG | 74 (10.3) | 114 (11.1) | 1.30 | 0.91–1.85 |  |
| Any variants | AA+AG | 312 (43.5) | 394 (38.5) | 1.72 | 1.35–2.18 |  |
|  | GG | 136 (18.9) | 121 (11.8) | 2.33 | 1.70–3.18 | < 0.0001 |
| MC1R*rs935053 |  |  |  |  |  | 0.85 |
| MC1R |  |  |  |  |  |  |
| No variants | AA+AG | 196 (27.3) | 395 (38.6) | Reference |  |  |
|  | GG | 74 (10.3) | 114 (11.1) | 1.30 | 0.91–1.85 |  |
| No RHC variants | AA+AG | 201 (28) | 292 (28.5) | 1.51 | 1.16–1.95 |  |
|  | GG | 80 (11.1) | 85 (8.3) | 1.97 | 1.37–2.84 |  |
| RHC variants | AA+AG | 111 (15.5) | 102 (10) | 2.32 | 1.66–3.24 |  |
|  | GG | 56 (7.8) | 36 (3.5) | 3.17 | 1.97–5.10 | < 0.0001 |
| MC1R*rs935053 |  |  |  |  |  | 0.99 |
|  | **rs10757257** |  |  |  |  |  |
| MC1R |  |  |  |  |  |  |
| No variants | AA+GA | 155 (21.6) | 332 (32.5) | Reference |  |  |
|  | GG | 115 (16)) | 177 (17.3) | 1.36 | 0.99–1.87 |  |
| Any variants | AA+GA | 269 (37.5) | 323 (31.6) | 1.89 | 1.45–2.46 |  |
|  | GG | 179 (24.9) | 190 (18.6) | 2.12 | 1.58–2.84 | < 0.0001 |
| MC1R*rs10757257 |  |  |  |  |  | 0.36 |
| MC1R |  |  |  |  |  |  |
| No variants | AA+GA | 155 (21.6) | 332 (32.5) | Reference |  |  |
|  | GG | 115 (16)) | 177 (17.3) | 1.36 | 0.99–1.87 |  |
| No RHC variants | AA+GA | 173 (24.1) | 246 (24.1) | 1.61 | 1.21–2.14 |  |
|  | GG | 108 (15) | 130 (12.7) | 1.90 | 1.36–2.64 |  |
| RHC variants | AA+GA | 96 (13.4) | 77 (7.5) | 2.78 | 1.91–4.03 |  |
|  | GG | 71 (9.9) | 60 (5.9) | 2.60 | 1.72–3.93 | < 0.0001 |
| MC1R*rs10757257 |  |  |  |  |  | 0.44 |
|  | **rs7023329** |  |  |  |  |  |
| MC1R |  |  |  |  |  |  |
| No variants | GG+AG | 188 (26.2) | 377 (36.8) | Reference |  |  |
|  | AA | 82 (11.4) | 133 (13) | 1.27 | 0.90–1.78 |  |
| Any variants | GG+AG | 321 (44.7) | 386 (37.7) | 1.81 | 1.42–2.30 |  |
|  | AA | 127 (17.7) | 129 (12.6) | 2.06 | 1.50–2.82 | < 0.0001 |
| MC1R*rs7023329 |  |  |  |  |  | 0.64 |
| MC1R |  |  |  |  |  |  |
| No variants | GG+AG | 188 (26.2) | 377 (36.8) | Reference |  |  |
|  | AA | 82 (11.4) | 133 (13) | 1.27 | 0.90–1.78 |  |
| No RHC variants | GG+AG | 206 (28.7) | 288 (28.1) | 1.57 | 1.21–2.04 |  |
|  | AA | 75 (10.5) | 89 (8.7) | 1.77 | 1.22–2.56 |  |
| RHC variants | GG+AG | 115 (16) | 98 (9.6) | 2.49 | 1.78–3.49 |  |
|  | AA | 52 (7.2) | 40 (3.9) | 2.70 | 1.69–4.33 | < 0.0001 |
| MC1R*rs7023329 |  |  |  |  |  | 0.85 |
|  | **rs10811629** |  |  |  |  |  |
| MC1R |  |  |  |  |  |  |
| No variants | GG+AG | 156 (21.7) | 334 (32.8) | Reference |  |  |
|  | AA | 114 (15.9) | 172 (16.9) | 1.39 | 1.01–1.91 |  |
| Any variants | GG+AG | 277 (38.6) | 332 (32.6) | 1.92 | 1.48–2.49 |  |
|  | AA | 171 (23.8) | 181 (17.8) | 2.08 | 1.54–2.79 | < 0.0001 |
| MC1R*rs10811629 |  |  |  |  |  | 0.24 |
| MC1R |  |  |  |  |  |  |
| No variants | GG+AG | 156 (21.7) | 334 (32.8) | Reference |  |  |
|  | AA | 114 (15.9) | 172 (16.9) | 1.39 | 1.01–1.91 |  |
| No RHC variants | GG+AG | 180 (25.1) | 254 (24.9) | 1.65 | 1.25–2.20 |  |
|  | AA | 101 (14.1) | 122 (12) | 1.82 | 1.29–2.55 |  |
| RHC variants | GG+AG | 97 (13.5) | 78 (7.7) | 2.77 | 1.91–4.02 |  |
|  | AA | 70 (9.8) | 59 (5.8) | 2.61 | 1.73–3.95 | < 0.0001 |
| MC1R*rs10811629 |  |  |  |  |  | 0.36 |
|  | **rs1011970** |  |  |  |  |  |
| MC1R |  |  |  |  |  |  |
| No variants | GG+GT | 255 (35.6) | 500 (49.1) | Reference |  |  |
|  | TT | 14 (2) | 7 (0.7) | 3.05 | 1.18–7.90 |  |
| Any variants | GG+GT | 433 (60.5) | 503 (49.4) | 1.78 | 1.45–2.19 |  |
|  | TT | 14 (2) | 8 (0.8) | 3.67 | 1.44–9.37 | < 0.0001 |
| MC1R*rs1011970 |  |  |  |  |  | 0.56 |
| MC1R |  |  |  |  |  |  |
| No variants | GG+GT | 255 (35.6) | 500 (49.1) | Reference |  |  |
|  | TT | 14 (2) | 7 (0.7) | 3.05 | 1.18–7.90 |  |
| No RHC variants | GG+GT | 269 (37.2) | 367 (36.1) | 1.53 | 1.22–1.92 |  |
|  | TT | 11 (1.5) | 7 (0.7) | 3.53 | 1.27–9.79 |  |
| RHC variants | GG+GT | 164 (22.9) | 136 (13.4) | 2.45 | 1.84–3.26 |  |
|  | TT | 3 (0.4) | 1 (0.1) | 4.49 | 0.43–47.34 | < 0.0001 |
| MC1R*rs1011970 |  |  |  |  |  | 0.88 |
|  | **rs3088440** |  |  |  |  |  |
| MC1R |  |  |  |  |  |  |
| No variants | CC+CT | 226 (31.5) | 457 (45.3) | Reference |  |  |
|  | TT | 43 (6.0) | 47 (4.7) | 1.70 | 1.07–2.72 |  |
| Any variants | CC+CT | 392 (54.7) | 449 (44.5) | 1.85 | 1.49–2.31 |  |
|  | TT | 56 (7.8) | 55 (5.5) | 2.16 | 1.42–3.30 | < 0.0001 |
| MC1R*rs3088440 |  |  |  |  |  | 0.24 |
| MC1R |  |  |  |  |  |  |
| No variants | CC+CT | 226 (31.5) | 457 (45.3) | Reference |  |  |
|  | TT | 43 (6.0) | 47 (4.7) | 1.70 | 1.07–2.72 |  |
| No RHC variants | CC+CT | 247 (34.5) | 327 (32.4) | 1.62 | 1.27–2.06 |  |
|  | TT | 34 (4.7) | 43 (4.3) | 1.69 | 1.03–2.78 |  |
| RHC variants | CC+CT | 145 (20.2) | 122 (12.1) | 2.47 | 1.82–3.34 |  |
|  | TT | 22 (3.1) | 12 (1.2) | 3.80 | 1.80–8.00 | < 0.0001 |
| MC1R*rs3088440 |  |  |  |  |  | 0.36 |
|  | **rs2811710** |  |  |  |  |  |
| MC1R |  |  |  |  |  |  |
| No variants | CC+CT | 220 (30.7) | 436 (42.8) | Reference |  |  |
|  | TT | 50 (7.0) | 72 (7.1) | 1.36 | 0.90–2.05 |  |
| Any variants | CC+CT | 378 (52.7) | 448 (44) | 1.05 | 1.04–1.06 |  |
|  | TT | 69 (9.6) | 62 (6.1) | 2.52 | 1.69–3.75 | < 0.0001 |
| MC1R*rs2811710 |  |  |  |  |  | 0.85 |
| MC1R |  |  |  |  |  |  |
| No variants | CC+CT | 220 (30.7) | 436 (42.8) | Reference |  |  |
|  | TT | 50 (7.0) | 72 (7.1) | 1.36 | 0.90–2.05 |  |
| No RHC variants | CC+CT | 238 (33.2) | 329 (32.3) | 1.52 | 1.19–1.94 |  |
|  | TT | 42 (5.9) | 43 (4.2) | 2.24 | 1.39–3.61 |  |
| RHC variants | CC+CT | 140 (19.5) | 119 (11.7) | 2.41 | 1.77–3.28 |  |
|  | TT | 27 (3.8) | 19 (1.9) | 3.12 | 1.65–5.91 | < 0.0001 |
| MC1R*rs2811710 |  |  |  |  |  | 0.95 |
