# Supplementary material for: Variants at the 9p21 locus and melanoma risk
Source: BMC Cancer. 2013 Jul 2;13:325. doi: 10.1186/1471-2407-13-325 (PMC3702420; doi:10.1186/1471-2407-13-325)
Supplement: Additional file 5: Table S5 — Effect of interaction between the polymorphisms rs751173, rs4636294, rs2218220, rs1335510, rs1341866, rs935053, rs10757257, rs7023329, rs10811629, rs1011970, rs3088440, rs2811710 and phenotypic traits on melanoma risk. [file 1471-2407-13-325-S5.docx]

Additional file 5. Effect of interaction between the polymorphisms rs751173, rs4636294, rs2218220, rs1335510, rs1341866, rs935053, rs10757257, rs7023329, rs10811629, rs1011970, rs3088440 , rs2811710 and phenotypic traits on melanoma risk

| **Variable** | **SNP** | **Cases (%)** | **Controls (%)** | **OR** | **95% CI** | **P value** |
| --- | --- | --- | --- | --- | --- | --- |
|  | **rs751173** |  |  |  |  |  |
| Skin color |  |  |  |  |  |  |
| I | TT+TC | 21 (2.8) | 46 (4.6) | 2.56 | 1.01–6.49 |  |
|  | CC | 9 (1.2) | 11 (1) | 4.84 | 1.49–15.74 |  |
| II | TT+TC | 196 (26.3) | 176 (17.7) | 6.83 | 3.11–15.02 |  |
|  | CC | 62 (8.3) | 43 (4.3) | 9.22 | 3.89–21.86 |  |
| III | TT+TC | 204 (27.4) | 359 (36) | 3.39 | 1.56–7.39 |  |
|  | CC | 66 (8.9) | 80 (8) | 4.91 | 3.89–21.86 |  |
| IV | TT+TC | 144 (19.3) | 175 (17.6) | 4.50 | 2.04–9.95 |  |
|  | CC | 32 (4.3) | 41 (4.1) | 4.56 | 1.86–11.21 |  |
| V | TT+TC | 8 (1.1) | 52 (5.2) | Reference |  |  |
|  | CC | 4 (0.5) | 13 (1.3) | 1.68 | 0.42–6.76 | < 0.0001 |
| Skin*rs751173 |  |  |  |  |  | 0.79 |
| Hair color |  |  |  |  |  |  |
| Black brown | TT+TC | 434 (58.8) | 850 (74.9) | Reference |  |  |
|  | CC | 131 (17.8) | 202 (17.8) | 1.28 | 0.99–1.66 |  |
| Red blond | TT+TC | 134 (18.2) | 70 (6.2) | 3.74 | 2.71–5.18 |  |
|  | CC | 39 (5.3) | 13 (1.2) | 6.10 | 3.13–11.86 | < 0.0001 |
| Hair*rs751173 |  |  |  |  |  | 0.54 |
| Eye color |  |  |  |  |  |  |
| Black brown | TT+TC | 344 (46.6) | 670 (59.5) | Reference |  |  |
|  | CC | 111 (15) | 159 (14.1) | 1.32 | 0.99–1.76 |  |
| Blue green | TT+TC | 224 (30.3) | 240 (21.3) | 1.78 | 1.41–2.25 |  |
|  | CC | 60 (8.1) | 57 (5.1) | 2.18 | 1.46–3.26 | < 0.0001 |
| Eye*rs751173 |  |  |  |  |  | 0.78 |
|  | **rs4636294** |  |  |  |  |  |
| Phototype |  |  |  |  |  |  |
| I | GG+GA | 18 (2.4) | 38 (3.8) | 2.17 | 0.85–5.59 |  |
|  | AA | 12 (1.6) | 19 (1.9) | 3.24 | 1.15–9.17 |  |
| II | GG+GA | 177 (23.8) | 172 (17.3) | 5.36 | 2.49–11.55 |  |
|  | AA | 81 (10.9) | 47 (4.7) | 9.16 | 4.00–28.99 |  |
| III | GG+GA | 191 (25.6) | 345 (34.6) | 2.82 | 1.32–6.02 |  |
|  | AA | 78 (10.5) | 95 (9.5) | 4.01 | 1.81–8.92 |  |
| IV | GG+GA | 129 (17.3) | 173 (17.4) | 3.51 | 1.62–7.60 |  |
|  | AA | 47 (6.3) | 43 (4.3) | 5.00 | 2.12–11.72 |  |
| V | GG+GA | 9 (1.2) | 47 (4.7) | Reference |  |  |
|  | AA | 3 (0.4) | 18 (1.8) | 0.89 | 0.21–3.74 | < 0.0001 |
| Photoytpe*rs4636294 |  |  |  |  |  | 0.92 |
| Hair color |  |  |  |  |  |  |
| Black brown | GG+GA | 406 (55.1) | 810 (71.3) | Reference |  |  |
|  | AA | 158 (21.4) | 243 (21.4) | 1.26 | 0.99–1.61 |  |
| Red blond | GG+GA | 114 (15.5) | 65 (5.7) | 3.52 | 2.50–4.95 |  |
|  | AA | 59 (8) | 18 (1.6) | 6.38 | 3.64–11.18 | < 0.0001 |
| Hair*rs4636294 |  |  |  |  |  | 0.30 |
| Eye color |  |  |  |  |  |  |
| Black brown | GG+GA | 319 (43.2) | 641 (56.9) | Reference |  |  |
|  | AA | 135 (18.3) | 189 (16.8) | 1.33 | 1.01–1.75 |  |
| Blue green | GG+GA | 201 (27.2) | 222 (19.7) | 1.76 | 1.37–2.24 |  |
|  | AA | 83 (11.3) | 75 (6.7) | 2.28 | 1.60–3.25 | < 0.0001 |
| Eye*rs4636294 |  |  |  |  |  | 0.92 |
|  | **rs2218220** |  |  |  |  |  |
| Phototype |  |  |  |  |  |  |
| I | TT+CT | 18 (2.4) | 40 (4) | 2.07 | 0.81–5.31 |  |
|  | CC | 12 (1.6) | 16 (1.6) | 3.80 | 1.32–10.95 |  |
| II | TT+CT | 177 (23.7) | 171 (17.2) | 5.38 | 2.50–11.60 |  |
|  | CC | 81 (10.9) | 48 (4.8) | 8.98 | 3.92–20.53 |  |
| III | TT+CT | 192 (25.7) | 347 (34.8) | 2.81 | 1.32–6.00 |  |
|  | CC | 78 (10.5) | 94 (9.4) | 4.05 | 1.82–9.00 |  |
| IV | TT+CT | 130 (17.4) | 172 (17.3) | 3.55 | 1.64–7.69 |  |
|  | CC | 46 (6.2) | 43 (4.3) | 4.86 | 2.07–11.44 |  |
| V | TT+CT | 9 (1.2) | 47 (4.7) | Reference |  |  |
|  | CC | 3 (0.4) | 18 (1.8) | 0.89 | 0.21–3.74 | < 0.0001 |
| Phototype*rs2218220 |  |  |  |  |  | 0.90 |
| Hair color |  |  |  |  |  |  |
| Black brown | TT+CT | 408 (55.3) | 813 (71.6) | Reference |  |  |
|  | CC | 157 (21.3) | 239 (21.1) | 1.28 | 1.00–1.63 |  |
| Red blond | TT+CT | 114 (15.5) | 65 (5.7) | 3.52 | 2.50–4.95 |  |
|  | CC | 59 (8) | 18 (1.6) | 6.37 | 3.64–11.17 | < 0.0001 |
| Hair color |  |  |  |  |  | < 0.0001 |
| rs2218220 |  |  |  |  |  | 0.02 |
| Hair and rs2218220 |  |  |  |  |  | 0.31 |
| Eye color |  |  |  |  |  |  |
| Black brown | TT+CT | 321 (43.4) | 643 (57.1) | Reference |  |  |
|  | CC | 134 (18.1) | 186 (16.5) | 1.34 | 1.02–1.76 |  |
| Blue green | TT+CT | 201 (27.2) | 223 (19.8) | 1.74 | 1.36–2.22 |  |
|  | CC | 83 (11.2) | 74 (6.6) | 2.31 | 1.62–3.31 | < 0.0001 |
| Eye*rs2218220 |  |  |  |  |  | 0.98 |
|  | **rs935053** |  |  |  |  |  |
| Phototype |  |  |  |  |  |  |
| I | AA+AG | 18 (2.4) | 40 (4) | 2.07 | 0.81–5.31 |  |
|  | GG | 12 (1.6) | 17 (1.7) | 3.60 | 1.26–10.31 |  |
| II | AA+AG | 176 (23.6) | 171 (17.2) | 5.35 | 2.48–11.5 |  |
|  | GG | 82 (11) | 47 (4.7) | 9.32 | 4.07–21.34 |  |
| III | AA+AG | 192 (25.8) | 348 (34.9) | 2.82 | 1.32–6.02 |  |
|  | GG | 77 (10.3) | 93 (9.3) | 3.94 | 1.77–8.77 |  |
| IV | AA+AG | 130 (17.5) | 177 (17.8) | 3.46 | 1.60–7.50 |  |
|  | GG | 46 (6.2) | 39 (3.9) | 5.33 | 2.25–12.62 |  |
| V | AA+AG | 9 (1.2) | 47 (4.7) | Reference |  |  |
|  | GG | 3 (0.4) | 18 (1.8) | 0.89 | 0.21–3.74 | < 0.0001 |
| Phototype*rs935053 |  |  |  |  |  | 0.88 |
| Hair color |  |  |  |  |  |  |
| Black brown | AA+AG | 406 (55.1) | 819 (72.1) | Reference |  |  |
|  | GG | 158 (21.4) | 234 (20.6) | 1.31 | 1.02–1.67 |  |
| Red blond | AA+AG | 114 (15.5) | 65 (5.7) | 3.55 | 2.52–4.99 |  |
|  | GG | 59 (8) | 18 (1.6) | 6.43 | 3.67–11.26 | < 0.0001 |
| Hair* rs935053 |  |  |  |  |  | 0.35 |
| Eye color |  |  |  |  |  |  |
| Black brown | AA+AG | 320 (43.4) | 649 (57.6) | Reference |  |  |
|  | GG | 134 (18.2) | 181 (16.1) | 1.38 | 1.05–1.82 |  |
| Blue green | AA+AG | 201 (27.2) | 224 (19.9) | 1.77 | 1.38–2.25 |  |
|  | GG | 83 (11.3) | 73 (6.5) | 2.32 | 1.63–3.32 | < 0.0001 |
| Eye*rs935053 |  |  |  |  |  | 0.84 |
|  | **rs1335510** |  |  |  |  |  |
| Phototype |  |  |  |  |  |  |
| I | GG+TG | 11 (1.5) | 32 (3.2) | 2.33 | 0.70–7.71 |  |
|  | TT | 19 (2.6) | 24 (2.4) | 6.33 | 2.02–19.86 |  |
| II | GG+TG | 145 (19.5) | 145 (4.6) | 7.91 | 2.95–21.25 |  |
|  | TT | 113 (15.2) | 73 (7.4) | 13.08 | 4.76–35.85 |  |
| III | GG+TG | 162 (21.8) | 282 (28.5) | 4.54 | 1.70–12.08 |  |
|  | TT | 106 (14.3) | 155 (15.6) | 5.20 | 1.93–14.03 |  |
| IV | GG+TG | 108 (14.5) | 150 (15.1) | 5.24 | 1.94–14.14 |  |
|  | TT | 68 (9.1) | 66 (6.7) | 7.37 | 2.65–20.46 |  |
| V | GG+TG | 5 (0.7) | 40 (4) | Reference |  |  |
|  | TT | 7 (0.9) | 24 (2.4) | 2.36 | 0.65–8.53 | < 0.0001 |
| Phototype*rs1335510 |  |  |  |  |  | 0.33 |
| Hair color |  |  |  |  |  |  |
| Black brown | GG+TG | 333 (45.2) | 683 (60.4) | Reference |  |  |
|  | TT | 231 (31.4) | 365 (32.3) | 1.29 | 1.04–1.61 |  |
| Red blond | GG+TG | 94 (12.8) | 50 (4.4) | 3.78 | 2.58–5.55 |  |
|  | TT | 78 (10.6) | 32 (2.8) | 5.10 | 3.25–8.00 | < 0.0001 |
| Hair*rs1335510 |  |  |  |  |  | 0.89 |
| Eye color |  |  |  |  |  |  |
| Black brown | GG+TG | 262 (35.6) | 546 (48.7) | Reference |  |  |
|  | TT | 192 (26.1) | 281 (25.1) | 1.39 | 1.08–1.78 |  |
| Blue green | GG+TG | 165 (22.4) | 177 (15.8) | 1.90 | 1.45–2.49 |  |
|  | TT | 118 (16) | 117 (10.4) | 2.11 | 1.55–2.86 | < 0.0001 |
| Eye*rs1335510 |  |  |  |  |  | 0.30 |
|  | **rs1341866** |  |  |  |  |  |
| Phototype |  |  |  |  |  |  |
| I | CC+TC | 11 (1.5) | 33 (3.3) | 2.32 | 0.70–7.67 |  |
|  | TT | 19 (2.6) | 24 (2.4) | 6.46 | 2.07–20.22 |  |
| II | CC+TC | 149 (20) | 146 (14.7) | 8.30 | 3.10–22.23 |  |
|  | TT | 109 (14.6) | 71 (7.1) | 13.07 | 4.78–35.77 |  |
| III | CC+TC | 166 (22.3) | 287 (28.8) | 4.67 | 1.76–12.39 |  |
|  | TT | 104 (13.9) | 153 (14.4) | 5.31 | 1.97–14.28 |  |
| IV | CC+TC | 109 (14.6) | 154 (15.5) | 5.28 | 1.96–14.19 |  |
|  | TT | 67 (9) | 62 (6.2) | 7.84 | 2.83–21.80 |  |
| V | CC+TC | 5 (0.7) | 41 (4.1) | Reference |  |  |
|  | TT | 7 (0.9) | 24 (2.4) | 2.41 | 0.67–8.69 | < 0.0001 |
| Phototype*rs1341866 |  |  |  |  |  | 0.32 |
| Hair color |  |  |  |  |  |  |
| Black brown | CC+TC | 339 (45.9) | 695 (61.3) | Reference |  |  |
|  | TT | 226 (30.6) | 357 (31.5) | 1.28 | 1.03–1.60 |  |
| Red blond | CC+TC | 97 (13.1) | 50 (4.4) | 3.91 | 2.67–5.72 |  |
|  | TT | 76 (10.3) | 32 (2.8) | 4.96 | 3.16–7.79 | < 0.0001 |
| Hair* rs1341866 |  |  |  |  |  | 0.97 |
| Eye color |  |  |  |  |  |  |
| Black brown | CC+TC | 268 (35.3) | 556 (49.4) | Reference |  |  |
|  | TT | 187 (25.3) | 274 (24.4) | 1.38 | 1.08–1.77 |  |
| Blue green | CC+TC | 168 (22.7) | 180 (16) | 1.91 | 1.46–2.50 |  |
|  | TT | 116 (15.7) | 115 (10.2) | 2.08 | 1.53–2.83 | < 0.0001 |
| Eye*rs1341866 |  |  |  |  |  | 0.28 |
|  | **rs10757257** |  |  |  |  |  |
| Phototype |  |  |  |  |  |  |
| I | AA+GA | 10 (1.3) | 33 (3.2) | 2.41 | 0.67–8.68 |  |
|  | GG | 20 (2.7) | 24 (2.4) | 7.80 | 2.32–26.20 |  |
| II | AA+GA | 145 (19.4) | 146 (14.7) | 9.27 | 3.17–27.08 |  |
|  | GG | 113 (15.2) | 71 (7.1) | 15.62 | 5.25–46.48 |  |
| III | AA+GA | 167 (22.4) | 282 (28.3) | 5.54 | 1.91–16.05 |  |
|  | GG | 103 (13.8) | 158 (15.9) | 5.79 | 1.97–16.98 |  |
| IV | AA+GA | 110 (14.8) | 152 (15.3) | 6.18 | 2.11–18.13 |  |
|  | GG | 66 (8.9) | 64 (6.4) | 8.65 | 2.86–26.12 |  |
| V | AA+GA | 4 (0.5) | 40 (4) | Reference |  |  |
|  | GG | 8 (1.1) | 25 (2.5) | 2.88 | 0.76–10.89 | < 0.0001 |
| Phototype*rs10757257 |  |  |  |  |  | 0.09 |
| Hair color |  |  |  |  |  |  |
| Black brown | AA+GA | 336 (45.5) | 685 (60.4) | Reference |  |  |
|  | GG | 229 (31) | 366 (32.3) | 1.25 | 1.00–1.55 |  |
| Red blond | AA+GA | 96 (13) | 51 (4.5) | 3.73 | 2.55–5.44 |  |
|  | GG | 77 (10.4) | 32 (2.8) | 5.04 | 3.21–7.90 | < 0.0001 |
| Hair*rs10757257 |  |  |  |  |  | 0.79 |
| Eye color |  |  |  |  |  |  |
| Black brown | AA+GA | 266 (36) | 549 (48.8) | Reference |  |  |
|  | GG | 189 (25.6) | 281 (25) | 1.33 | 1.04–1.70 |  |
| Blue green | AA+GA | 166(22.5) | 178 (15.8) | 1.88 | 1.43–2.46 |  |
|  | GG | 117 (10.4) | 118 (16) | 2.06 | 1.52–2.81 | < 0.0001 |
| Eye*rs10757257 |  |  |  |  |  | 0.38 |
|  | **rs7023329** |  |  |  |  |  |
| Phototype |  |  |  |  |  |  |
| I | GG+AG | 18 (2.4) | 38 (3.8) | 2.74 | 1.01–7.46 |  |
|  | AA | 12 (1.6) | 19 (1.9) | 4.02 | 1.34–12.05 |  |
| II | GG+AG | 179 (24) | 172 (17.3) | 6.70 | 2.90–15.50 |  |
|  | AA | 79 (10.6) | 47 (4.7) | 11.45 | 4.68–28.03 |  |
| III | GG+AG | 187 (25.1) | 326 (32.7) | 3.64 | 1.59–8.36 |  |
|  | AA | 83 (11.1) | 114 (11.4) | 4.46 | 1.89–10.55 |  |
| IV | GG+AG | 131 (17.6) | 170 (17.1) | 4.54 | 1.95–10.55 |  |
|  | AA | 45 (6) | 46 (4.6) | 5.55 | 2.22–13.88 |  |
| V | GG+AG | 7 (0.9) | 47 (4.7) | Reference |  |  |
|  | AA | 5 (0.7) | 18 (1.8) | 1.74 | 0.47–6.42 | < 0.0001 |
| Phototype*rs7023329 |  |  |  |  |  | 0.79 |
| Hair color |  |  |  |  |  |  |
| Black brown | GG+AG | 402 (54.5) | 792 (69.7) | Reference |  |  |
|  | AA | 163 (22.1) | 261 (23) | 1.20 | 0.95–1.53 |  |
| Red blond | GG+AG | 116 (15.7) | 62 (5.5) | 3.64 | 2.58–5.14 |  |
|  | AA | 57 (7.7) | 21 (1.9) | 5.46 | 3.20–9.32 | < 0.0001 |
| Hair*rs7023329 |  |  |  |  |  | 0.51 |
| Eye color |  |  |  |  |  |  |
| Black brown | GG+AG | 316 (42.8) | 631 (56) | Reference |  |  |
|  | AA | 139 (18.8) | 200 (17.8) | 1.32 | 1.01–1.72 |  |
| Blue green | GG+AG | 202 (27.3) | 212 (18.8) | 1.85 | 1.45–2.37 |  |
|  | AA | 82 (11.1) | 84 (7.5) | 1.98 | 1.40–2.80 | < 0.0001 |
| Eye*rs7023329 |  |  |  |  |  | 0.37 |
|  | **rs10811629** |  |  |  |  |  |
| Phototype |  |  |  |  |  |  |
| I | GG+AG | 10 (1.3) | 33 (3.3) | 2.35 | 0.65–8.45 |  |
|  | AA | 20 (2.7) | 24 (2.4) | 8.43 | 2.51–28.33 |  |
| II | GG+AG | 151 (20.2) | 152 (15.3) | 9.70 | 3.33–28.28 |  |
|  | AA | 107 (14.3) | 65 (6.6) | 15.93 | 5.34–47.54 |  |
| III | GG+AG | 169 (22.7) | 285 (28.7) | 5.72 | 1.98–16.57 |  |
|  | AA | 101 (13.5) | 153 (15.4) | 5.83 | 1.99–17.11 |  |
| IV | GG+AG | 110 (14.8) | 143 (14.4) | 6.73 | 2.30–19.74 |  |
|  | AA | 66 (8.9) | 72 (7.3) | 7.79 | 2.59–23.43 |  |
| V | GG+AG | 4 (0.5) | 40 (4) | Reference |  |  |
|  | AA | 8 (1.1) | 25 (2.5) | 3.04 | 0.80–11.47 | < 0.0001 |
| **Phototype*rs10811629** |  |  |  |  |  | **0.05** |
| Hair color |  |  |  |  |  |  |
| Black brown | GG+AG | 346 (46.9) | 695 (61.5) | Reference |  |  |
|  | AA | 219 (29.7) | 353 (31.2) | 1.17 | 0.94–1.47 |  |
| Red blond | GG+AG | 94 (12.7) | 50 (4.4) | 3.54 | 2.42–5.18 |  |
|  | AA | 79 (10.7) | 33 (2.9) | 5.02 | 3.22–7.83 | < 0.0001 |
| Hair*rs10811629 |  |  |  |  |  | 0.53 |
| Eye color |  |  |  |  |  |  |
| Black brown | GG+AG | 280 (37.9) | 543 (48.4) | Reference |  |  |
|  | AA | 175 (23.7) | 283 (25.20 | 1.12 | 0.88–1.44 |  |
| Blue green | GG+AG | 160 (21.7) | 193 (17.2) | 1.58 | 1.21–2.06 |  |
|  | AA | 124 (16.8) | 103 (9.2) | 2.27 | 1.66–3.11 | < 0.0001 |
| Eye and rs10811629 |  |  |  |  |  | 0.25 |
|  | **rs1011970** |  |  |  |  |  |
| Phototype |  |  |  |  |  |  |
| I | GG+GT | 29 (3.9) | 53 (5.4) | 2.80 | 1.25–6.28 |  |
|  | TT | 1 (0.1)) | 3 (0.3) | 1.47 | 0.13–16.45 |  |
| II | GG+GT | 248 (33.4) | 214 (21.6) | 6.37 | 3.22–12.63 |  |
|  | TT | 10 (1.4) | 4 (0.4) | 13.55 | 3.42–53.75 |  |
| III | GG+GT | 257 (34.6) | 433 (43.7) | 3.17 | 1.61–6.23 |  |
|  | TT | 12 (1.6) | 5 (0.5) | 10.58 | 2.91–38.47 |  |
| IV | GG+GT | 167 (22.5) | 212 (21.4) | 3.91 | 1.96–7.79 |  |
|  | TT | 6 (0.8) | 3 (0.3) | 10.03 | 2.04–42.29 |  |
| V | GG+GT | 11 (1.5) | 62 (6.3) | Reference |  |  |
|  | TT | 1 (0.1) | 2 (0.2) | 2.01 | 0.15–26.16 | < 0.0001 |
| Phototype*rs1011970 |  |  |  |  |  | 0.74 |
| Hair color |  |  |  |  |  |  |
| Black brown | GG+GT | 539 (73.4) | 1030 (91.2) | Reference |  |  |
| Red blond | GG+GT | 165 (22.5) | 82 (7.3) | 3.85 | 2.86–5.19 |  |
| Black brown/ Red blond* | TT | 30 (4.1) | 17 (1.5) | 2.99 | 1.59–5.65 | < 0.0001 |
| Hair*rs1011970 |  |  |  |  |  | 0.98 |
| Eye color |  |  |  |  |  |  |
| Black brown | GG+GT | 431 (58.6) | 811 (72.4) | Reference |  |  |
|  | TT | 20 (2.7) | 13 (1.2) | 2.47 | 1.17–5.22 |  |
| Blue green | GG+GT | 274 (37.3) | 292 (26.1) | 1.76 | 1.42–2.17 |  |
|  | TT | 10 (1.4) | 4 (0.4) | 4.29 | 1.29–14.26 | < 0.0001 |
| Eye*rs1011970 |  |  |  |  |  | 0.99 |
|  | **rs3088440** |  |  |  |  |  |
| Phototype |  |  |  |  |  |  |
| I | CC+CT | 27 (3.6) | 54 (5.5) | 3.42 | 1.39–8.38 |  |
|  | TT | 3 (0.4) | 3 (0.3) | 5.87 | 0.91–37.85 |  |
| II | CC+CT | 225 (30.2) | 183 (18.6) | 9.07 | 4.14–19.89 |  |
|  | TT | 32 (4.3) | 30 (3.1) | 7.61 | 3.04–19.09 |  |
| III | CC+CT | 226 (30.3) | 402 (40.9) | 3.99 | 1.83–8.67 |  |
|  | TT | 44 (5.9) | 35 (3.6) | 8.80 | 3.62–21.42 |  |
| IV | CC+CT | 150 (20.1) | 188 (19.1) | 5.24 | 2.38–11.54 |  |
|  | TT | 26 (3.5) | 22 (2.2) | 8.01 | 3.05–21.04 |  |
| V | CC+CT | 8 (1.1) | 57 (5.8) | Reference |  |  |
|  | TT | 4 (0.5) | 8 (0.8) | 4.30 | 1.01–18.38 | < 0.0001 |
| Phototype*rs3088440 |  |  |  |  |  | 0.08 |
| Hair color |  |  |  |  |  |  |
| Black brown | CC+CT | 476 (64.6) | 936 (83.7) | Reference |  |  |
|  | TT | 88 (11.9) | 102 (9.1) | 1.74 | 1.26–2.40 |  |
| Red blond | CC+CT | 152 (20.6) | 72 (6.4) | 4.30 | 3.14–5.89 |  |
|  | TT | 21 (2.9) | 8 (0.7) | 4.04 | 1.71–9.51 | < 0.0001 |
| Hair*rs3088440 |  |  |  |  |  | 0.21 |
| Eye color |  |  |  |  |  |  |
| Black brown | CC+CT | 400 (54.2) | 741 (66.9) | Reference |  |  |
|  | TT | 55 (7.5) | 76 (6.9) | 1.36 | 0.93–2.01 |  |
| Blue green | CC+CT | 230 (31.2) | 260 (23.5) | 1.66 | 1.32–2.07 |  |
|  | TT | 53 (7.2) | 30 (2.7) | 3.08 | 1.90–5.00 | < 0.0001 |
| Eye*rs3088440 |  |  |  |  |  | 0.33 |
|  | **rs2811710** |  |  |  |  |  |
| Phototype |  |  |  |  |  |  |
| I | CC+CT | 25 (3.4) | 51 (5.1) | 2.55 | 1.09–5.97 |  |
|  | TT | 5 (0.7) | 6 (0.6) | 4.19 | 1.00–17.61 |  |
| II | CC+CT | 209 (28.1) | 184 (18.5) | 6.37 | 3.10–13.09 |  |
|  | TT | 48 (6.4) | 35 (3.5) | 7.70 | 3.36–17.62 |  |
| III | CC+CT | 231 (31) | 379 (38.1) | 3.28 | 1.61–6.67 |  |
|  | TT | 39 (5.2) | 58 (5.8) | 3.84 | 1.71–8.64 |  |
| IV | CC+CT | 141 (18.9) | 189 (19) | 3.71 | 1.79–7.67 |  |
|  | TT | 35 (4.7) | 27 (2.7) | 6.95 | 2.91–16.58 |  |
| V | CC+CT | 10 (1.3) | 57 (5.7) | Reference |  |  |
|  | TT | 2 (0.3) | 8 (0.8) | 1.32 | 0.24–7.40 | < 0.0001 |
| Phototype*rs2811710 |  |  |  |  |  | 0.76 |
| Hair color |  |  |  |  |  |  |
| Black brown | CC+CT | 466 (63.2) | 905 (80) | Reference |  |  |
|  | TT | 99 (13.4) | 143 (12.6) | 1.42 | 1.06–1.90 |  |
| Red blond | CC+CT | 143 (19.4) | 75 (6.6) | 3.76 | 2.75–5.15 |  |
|  | TT | 29 (3.9) | 8 (0.7) | 6.95 | 3.05–15.85 | < 0.0001 |
| Hair*rs2811710 |  |  |  |  |  | 0.57 |
| Eye color |  |  |  |  |  |  |
| Black brown | CC+CT | 371 (50.3) | 717 (64) | Reference |  |  |
|  | TT | 84 (11.4) | 107 (9.6) | 1.56 | 1.12–2.17 |  |
| Blue green | CC+CT | 239 (32.4) | 255 (22.8) | 1.81 | 1.44–2.27 |  |
|  | TT | 44 (6) | 41 (3.7) | 2.18 | 1.37–3.48 | < 0.0001 |
| Eye*rs2811710 |  |  |  |  |  | 0.39 |
